# Applying the Ottawa Charter to evaluate health literacy outcomes of the Little Aussie Bugs course for Australian early childhood educators

**DOI:** 10.1093/heapro/daag087

**Published:** 2026-07-06

**Authors:** Amelia Ruscoe, Elizabeth J Cook, Eleanor K O’Brien, Leesa Costello, Gillian Kirk, Ros Sambell, Theresa Rayfield, Amanda Devine, Ruth Wallace

**Affiliations:** School of Education, Edith Cowan University, 270 Joondalup Drive, Joondalup, 6027, Perth, Western Australia, Australia; Nutrition & Health Innovation Research Institute, Edith Cowan University, 270 Joondalup Drive, Joondalup, 6027, Perth, Western Australia, Australia; School of Education, Faculty of Creative Industries, Education, Social Justice, Queensland University of Technology, Kelvin Grove, 4059, Brisbane, Queensland, Australia; School of Medical & Health Science, Edith Cowan University, 270 Joondalup Drive, Joondalup, 6027, Perth, Western Australia, Australia; Centre for Inclusive Education, Faculty of Creative Industries, Education, Social Justice, Queensland University of Technology, Kelvin Grove, 4059, Brisbane, Queensland, Australia; School of Medical & Health Science, Edith Cowan University, 270 Joondalup Drive, Joondalup, 6027, Perth, Western Australia, Australia; Centre for Precision Health, Edith Cowan University, 270 Joondalup Drive, Joondalup, 6027, Perth, Western Australia, Australia; Nutrition & Health Innovation Research Institute, Edith Cowan University, 270 Joondalup Drive, Joondalup, 6027, Perth, Western Australia, Australia; School of Medical & Health Science, Edith Cowan University, 270 Joondalup Drive, Joondalup, 6027, Perth, Western Australia, Australia; School of Education, Edith Cowan University, 270 Joondalup Drive, Joondalup, 6027, Perth, Western Australia, Australia; Nutrition & Health Innovation Research Institute, Edith Cowan University, 270 Joondalup Drive, Joondalup, 6027, Perth, Western Australia, Australia; School of Medical & Health Science, Edith Cowan University, 270 Joondalup Drive, Joondalup, 6027, Perth, Western Australia, Australia; School of Medical & Health Science, Edith Cowan University, 270 Joondalup Drive, Joondalup, 6027, Perth, Western Australia, Australia; Nutrition & Health Innovation Research Institute, Edith Cowan University, 270 Joondalup Drive, Joondalup, 6027, Perth, Western Australia, Australia; School of Medical & Health Science, Edith Cowan University, 270 Joondalup Drive, Joondalup, 6027, Perth, Western Australia, Australia; Nutrition & Health Innovation Research Institute, Edith Cowan University, 270 Joondalup Drive, Joondalup, 6027, Perth, Western Australia, Australia; School of Medical & Health Science, Edith Cowan University, 270 Joondalup Drive, Joondalup, 6027, Perth, Western Australia, Australia

**Keywords:** health literacy, early childhood education and care, dialogic reading, Ottawa Charter for Health Promotion, professional development, supportive environments, mixed methods evaluation

## Abstract

Early childhood offers an important opportunity to lay the foundations for lifelong health literacy. The Little Aussie Bugs (LAB) dialogic book series and online professional development (PD) course were designed to support Early Childhood Education and Care (ECEC) educators to embed health literacy concepts, including hygiene, healthy eating, and oral health, into everyday learning routines. This mixed methods study evaluated the course pilot and examined its contribution through the lens of the Ottawa Charter for Health Promotion—a framework comprising five interrelated action areas: building healthy public policy; creating supportive environments; strengthening community action; developing personal skills; and reorienting health services. Quantitative findings demonstrated statistically significant improvements in educators’ overall health literacy scores and confidence using dialogic reading to deliver health messages. Educators reported enhanced capability to support children’s understanding of health behaviors. Qualitative findings revealed educators’ experiences embedding learning and LAB resources into daily routines, strengthening their confidence, supporting communications with families, and aligning practice with policy and accreditation expectations. Applying the Charter sharpened research insights into how the intervention developed personal skills and created supportive environments, while also illuminating structural gaps in policy alignment, educator training, and equitable implementation. This evaluation demonstrates how applying the Charter can support analysis of digital, early-years health promotion initiatives and help identify system-level supports for their sustainability. It also shows the course is an accessible and scalable approach to embed health literacy across ECEC settings. Implications for early-years PD and health literacy practice are discussed through the lens of the Charter.

Contribution to Health Promotion

- Provides evidence that a brief, scalable online professional development course can strengthen health literacy practices in ECEC settings.
- Demonstrates how dialogic reading and educator professional learning can operationalize health promotion within everyday ECEC routines.
- Shows how the Ottawa Charter’s five action areas for Health Promotion can be applied as an analytic framework to evaluate a digital early-years health literacy intervention.
- Highlights how Charter-informed evaluation reveals system-level enablers and barriers influencing implementation, equity, and the sustainability of programs/interventions.

## Background

Health literacy in the early years lays the foundation for children’s lifelong health, well-being, and learning (Csima *et al*. 2024, Nash *et al*. 2024) and is defined by Nutbeam (2025) as skills enabling individuals to access, understand, and use information to make everyday health decisions. During early childhood, children begin to make sense of their world through language, relationships, and everyday routines (Selman and Dilworth-Bart 2024), making this a critical window for establishing foundational health concepts relating to personal hygiene, nutrition, and oral health. This developmental opportunity unfolds within contemporary environments that present significant challenges including pervasive commercial food marketing and the growing influence of digital and social media (Lafontaine *et al*. 2025) alongside incorrect and inconsistent health information (Denniss and Lindberg 2025). These environmental determinants shape what children learn about health and the types of messages they encounter (Clark *et al*. 2020).

Contemporary child health indicators in Australia highlight the urgency of strengthening early health literacy. Rising rates of children living with overweight and obesity, increasing prevalence of mental health concerns, and persistent inequities in oral health and nutrition are well-established predictors of adult morbidity and health system burden (Islam *et al*. 2023, Wake *et al*. 2025). The Australian National Preventive Health Policy 2021–30 identifies the early years as a priority for building children’s capacity to understand their bodies, health behaviors, and everyday health decisions (Department of Health 2021, Hoddinott *et al*. 2024). Strengthening early health literacy is, therefore, an equity-focused investment, with Early Childhood Education and Care (ECEC) settings offering a unique opportunity to reach children during a formative period.

Early-years educators are well placed to counter these influences by supporting health-promoting routines and modeling healthy behaviors (Noble *et al*. 2020). This responsibility is reinforced by national policy frameworks, including the Early Years Learning Framework V2.0 (EYLF2.0) and the National Quality Standard (NQS), particularly Quality Area 2: Children’s Health and Safety, which mandates ECEC services to intentionally promote children’s physical health, safety, and well-being (Australian Children’s Education and Care Quality Authority 2018, Australian Government Department of Education 2022). However, research consistently shows that many educators feel underprepared to teach health concepts, lack confidence in their own health literacy, and have limited access to pedagogically grounded training (Phillips and Fenech 2023). This capability gap restricts the potential of ECEC services to act as equitable and supportive settings for early health literacy development, positioning educator capability as a critical leverage point for improvement.

A cross-sector approach to health promotion is essential to support optimal child health (Watson and Neil 2025), and dialogic reading offers a pedagogical bridge between early learning and health promotion (Wall *et al*. 2022). Dialogic reading uses interactive, language-rich engagement with books to support children’s understanding of otherwise abstract health concepts, such as germs, bodily functions, or nutrition cues. The Little Aussie Bugs (LAB) book series was developed to leverage this approach by embedding health messages into simple storylines, familiar characters, and repeated catchphrases (Wallace *et al*. 2025a). Early piloting showed that impact depended on educators’ understanding of dialogic reading and their confidence applying health literacy concepts in practice, highlighting the need for aligned professional development (PD, Wallace *et al*. 2025a).

Although PD is critical for translating health policy expectations into confident, evidence-based practice, early-years educators often report limited access to time-efficient, pedagogically relevant training in health topics (Phillips and Fenech 2023), and commonly rely on informal information seeking, including internet searches, to fill knowledge gaps (Wallace *et al*. 2025a). While digital information is readily available, it is frequently inconsistent, commercially influenced, or poorly aligned with early childhood pedagogy (Denniss and Lindberg 2025), creating risks for both educator confidence and message quality. Online PD offers a promising mechanism to address these challenges by providing flexible, scalable access to evidence-based content that is explicitly tailored to educators’ roles and learning contexts. When designed to integrate theory, practical modeling, and reflective application, short online PD can support educators’ knowledge development, confidence, and capacity to embed health promotion within everyday practice (Barbati *et al*. 2025, Wallace *et al*. 2025b). This study evaluates a brief, structured online PD course (‘the course’) designed to support educators’ health literacy and use of dialogic reading in ECEC settings.

### The Little Aussie Bugs course

The course was designed to replicate the content of the face-to-face workshops delivered when the books were piloted and evaluated, as discussed in Wallace *et al*. (2025a). Our learnings from that study (evaluating the books) informed the content of the course evaluated in this study, which aimed to:

- support educators’ understanding of health literacy and dialogic reading in ECEC contexts;
- demonstrate practical use of the LAB books to promote health literacy; and
- provide opportunities for educators to document and reflect on applied learning.

An advisory group, comprised of public health nutritionists, health promotion and literacy experts, early-years educators, educational leaders, senior lecturers, and learning designers, was established to guide course content development, educational design, and review. This group ensured the course was evidence-based, pedagogically sound, and pitched at an appropriate educational level for early-years educators in ECEC settings nationwide.

The course, which continues to operate today, takes approximately 90 minutes to complete and is self-paced. It combines evidence-based information summaries, short readings, videos, reflective journal prompts, discussion boards, quizzes, and a glossary of terms. The content emphasizes dialogic reading principles and models how the LAB books can be integrated into everyday routines, transitions, and intentional teaching moments in ECEC settings.

Delivered through the Canvas learning management system, the course introduces educators to key health literacy concepts relevant to early childhood, including mealtimes, personal hygiene, oral health, and the role of language in shaping children’s understanding of health behaviors. Supplementary Appendix A summarizes the course design and shows how the content aligns with the ECEC curriculum, namely, the EYLF2.0 and NQS Quality Area 2.

The course prompts educators to consider how catchphrases, visuals, and book-based activities can be used to reinforce children’s understanding of health in developmentally responsive ways. Educators engage with other enrolled colleagues through online discussion boards, where they share ideas and examples of practice, and document their reflections in a personal journal built into the course (Supplementary Appendix B).

### Theoretical framework

The Ottawa Charter for Health Promotion, established in 1986, provides a widely used framework for understanding how health is created through action across multiple levels, rather than through individual behavior change alone (World Health Organization 2026). The Charter articulates five interrelated action areas: building healthy public policy; creating supportive environments; strengthening community action; developing personal skills; and reorienting health services. Together, these action areas emphasize the interaction between individual capability, everyday environments, community settings, and broader systems in shaping health outcomes. While foundational to health promotion scholarship (Fry and Zask 2017), the Charter has been infrequently applied to the evaluation of early-years health literacy interventions or digital professional learning. Applying the Charter in this study, therefore, offers a structured way to examine educator learning alongside the contextual, organizational, and system-level conditions that influence how health literacy initiatives are implemented and sustained in ECEC settings.

Evaluating such professional learning requires attention to educators’ perceptions of the resource’s usefulness, as well as capturing demonstrable changes in their content knowledge and confidence, and how they applied their learning from the course in everyday practice. Mixed methods approaches are particularly valuable in this context, enabling short-term learning outcomes to be assessed using validated measures while also capturing how educators interpret, adapt, and enact learning within diverse ECEC environments.

### Study aim

This study evaluates the short-term learning and practice outcomes of the LAB course, designed to build early-years educators’ health literacy and dialogic reading capability. The Ottawa Charter for Health Promotion was applied as the analytic framework to examine how these outcomes were enacted across individual, organizational, and system-level domains in ECEC.

## Method

### Study design

A sequential mixed methods design was used, incorporating quantitative (pre- and post-questionnaires) and qualitative (semistructured interviews) components (Creswell and Creswell 2023). All participating educators completed pre- and post-questionnaires to assess their health literacy and dialogic reading knowledge and confidence before and after the course (Supplementary Appendix C). To ensure participants were tested on health literacy knowledge development attributed to the course, the course content (specifically the glossary of terms) was mapped to the 16-item short version (HLQ-EU-16) of the validated European health literacy survey questionnaire (Pelikan *et al*. 2019). Supplementary Appendix D provides this mapping. The glossary (Supplementary Appendix E) was used for this mapping exercise as it offered the most robust representation of learning that could be acquired through the course.

On completion of the course, participants received print and digital versions of the LAB books, along with resources to support their translation of learning into educational practice. At the end of the post-course questionnaire, they were invited to express interest to participate in an individual interview to discuss their experiences of the course and use of LAB resources in their center. Supplementary Appendix F provides the structured interview guide. This mixed methods approach enabled participant knowledge and confidence to be assessed while also providing researchers with understandings into how participants’ e-learning was applied (Creswell and Creswell 2023).

### Ethical approval

Ethical approval was granted by the Edith Cowan University Human Research Ethics Committee (HREC no. 2023-04496-WALLACE). Written or verbal informed consent was obtained from all participants.

### Participant recruitment

A convenience sampling approach was used, drawing on the research team’s existing professional networks, which was appropriate given the limited funding available (Salkind 2022). Recruitment primarily occurred through four ECEC organizations in Western Australia, each of which nominated staff to participate, with additional recruitment undertaken via social media networks.

Eligible participants were early-years educators aged over 18 years and employed in Australia. Individuals who expressed interest but were not initially recruited were placed on a waitlist, from which ten additional participants were randomly selected.

In total, 68 educators were invited to participate, and 39 completed the course and both pre- and post-course questionnaires. Educators who indicated interest in a follow-up interview were subsequently contacted to arrange a mutually convenient time. All participants received information outlining the study purpose and procedures prior to participation.

### Quantitative data collection and analysis

The pre- and post-course questionnaires (Supplementary Appendix B) captured demographic characteristics, health literacy, dialogic reading knowledge and confidence, and post-course feedback. Together, the outcome measures provided insight into the educators’ learning, confidence to apply new practices, and their perceptions of the course’s acceptability and usefulness within ECEC contexts.

#### Health literacy

As mentioned previously, the HLQ-EU-16 was used to assess participants’ health literacy development pre- and post-course. This tool was selected because it is short and can be self-administered, thus minimizing participant burden. It measures comprehensive health literacy and has been validated across numerous populations (Pelikan *et al*. 2019).

Health literacy items were coded and summed using a 0–4 scale (0 = “very difficult” to 4 = “very easy”) to create a composite health literacy score (range 0–64). Linear mixed-effects models were applied to examine differences between pre- and post-course scores, with time as the predictor and participant as the random effect. Additional models assessed potential differences by highest qualification or years of experience. Ordinal logistic regression was used to analyze changes in response patterns for each health literacy item.

#### Dialogic reading

Four questions to assess dialogic reading knowledge were developed. The change in the total number of correct responses (0–4) pre- and post-course was analyzed using ordinal logistic regression. Logistic regression models examined the change in the probability of correctly answering each item.

#### Course satisfaction

The post-course questionnaire also included seven 5-point Likert-scale items assessing satisfaction and perceived usefulness, and an open-ended item seeking suggestions for improvement.

### Qualitative data collection and analysis

Individual semistructured interviews were conducted via Microsoft Teams and explored educators’ experiences of the course, their understanding and use of dialogic reading, and application of LAB resources within their centers. Interviews followed a structured guide (Supplementary Appendix F) and were audio-recorded and transcribed verbatim.

A codebook thematic analysis approach was used (Braun and Clarke 2021). Coding and theming data was iterative and refined through discussion between two researchers and then reviewed and validated by a third. Analysis focused on how educators described changes in their knowledge, confidence, routines, environments, and interactions with children, families, and colleagues following participation in the course, and how these experiences aligned with different dimensions of health promotion in ECEC settings. After codebook analysis and inter-rater discussion, interview themes were examined based on the Charter’s five strategic action areas.

Strategies to enhance the trustworthiness of the qualitative findings were embedded throughout the process—including researcher triangulation, peer debriefing, and transparent documentation of analytic decisions—supporting credibility, dependability, and confirmability (Ahmed 2024). Pseudonyms were used to preserve participant anonymity.

### Analytic framework: Ottawa Charter for Health Promotion

This mixed methods evaluation was informed by the Ottawa Charter for Health Promotion, which provides a contemporary, multilevel lens for understanding how digital, educator-focused interventions contribute to health promotion in early childhood settings. Although foundational to health promotion scholarship (Thomas *et al*. 2025), the Charter has, to the best of our knowledge, rarely been applied to digital PD or early-years health literacy interventions.

The Charter served as an interpretive framework with all five action areas relevant to the intended mechanisms of the course. Applying the Charter enabled analysis of how educator learning, pedagogical practice, and ECEC environments intersect to support young children’s developing health literacy whilst considering broader contextual factors influencing implementation, including equity, access, and alignment with organizational and system-level conditions. The Charter conceptualizes health promotion as action occurring across multiple, interrelated domains rather than through individual behavior change alone. In this study, the five action areas were interpreted in ways relevant to early childhood education and care contexts. “Building healthy public policy” refers to how educators and centers align health-promoting practices with regulatory, accreditation, and organizational requirements. “Creating supportive environments” captures the embedding of health literacy into everyday routines, physical spaces, and pedagogical practices. “Strengthening community action” reflects collaboration and knowledge sharing within and across centers and with families. “Developing personal skills” encompasses educators’ and children’s growing knowledge, confidence, and capacity to engage in health-promoting behaviors. Finally, “reorienting health services” is understood here as the positioning of ECEC settings and educators as community-based sites of preventive health promotion, rather than as formal health services.

Together, the quantitative and qualitative data provided a basis for examining the impact of the course and its contribution to the Charter, aligning health promotion in early childhood settings.

## Results

Of the 39 participants, most were from Western Australia (*n* = 16), with others located across Queensland (*n* = 7), Victoria (*n* = 6), New South Wales (*n* = 5), the Australian Capital Territory (*n* = 2), South Australia (*n* = 2), and Tasmania (*n* = 1). Participants worked across a range of roles, including early-years educators, early childhood teachers (ECTs), lead educators, center directors or managers, and owners. All participants were female, except for one male.

Qualifications varied, with 17 participants reporting they held or were working toward a diploma, and 18 holding or working toward an undergraduate or postgraduate degree. Most participants (*n* = 31) had more than 5 years’ experience in the ECEC sector, including 23 with over 10 years’ experience.

Quantitative analyses examined whether the course achieved its intended short-term learning outcomes by assessing changes in educators’ health literacy, dialogic reading knowledge, and confidence to use LAB resources following course completion. The full quantitative analyses and results are provided in Supplementary Appendix G. Together, these quantitative findings indicate that participation in the course was associated with measurable short-term learning gains in educators’ overall health literacy and dialogic reading knowledge, alongside high levels of perceived usefulness and confidence in applying LAB resources in practice.

Participants’ dialogic reading knowledge increased significantly on completion of the course. For each question, more people gave a correct response post- vs pre-course, and this change was significant for question 1 (Supplementary Appendix G, Table S1). The total number of correct responses across the four dialogic reading questions also increased significantly from pre- to post-course (mean score 2.69 pre-course vs. 3.10 post-course; *P* = 0.04; Supplementary Appendix G, Table S2).

Health literacy improved pre- to post-course. Individually, all health literacy questions showed an increase in the number of participants responding “very easy” or “easy” (Supplementary Appendix G, Figure S1), and this change was significant (*P* < 0.05) for 9 of the 16 questions (Supplementary Appendix G, Table S3). Composite health literacy scores (i.e. the total score across all questions) increased significantly, with the mean score 5.9 points higher post-course (mean score pre-course 46.5 vs post-course 52.4; *P* < 0.001; Supplementary Appendix G, Table S4). The improvement in composite health literacy scores remained significant if years of experience and the highest qualification of participants were included in the model, although neither of these factors was significantly associated with change in these scores (Supplementary Appendix G, Table S5).

Table 1 (below) shows that course feedback was positive, with 95% of participants reporting that it improved their confidence to use LAB resources at their center.

**Table 1:** Post-course feedback from participants (*n* = 39).

Variable	Much too long	Too long	About the right length	Too short	Much too short
The length of the online professional development was appropriate	0	2 (5%)	37 (95%)	0	0

Variable	Strongly agree	Somewhat agree	Neither agree nor disagree	Somewhat disagree	Strongly disagree
The content of the online professional development was easy to understand	36 (92%)	3 (8%)	0	0	0
I found the online professional development engaging	31 (79%)	8 (21%)	0	0	0
The online professional development improved my confidence to use the Little Aussie Bugs resources at the center	37 (95%)	2 (5%)	0	0	0
The online professional development improved my knowledge of health literacy	32 (82%)	6 (16%)	1 (2%)	0	0
The online professional development improved my knowledge about dialogic reading	32 (82%)	7 (18%)	0	0	0
The downloadable resources available from the online professional development were useful	33 (84%)	6 (16%)	0	0	0

Qualitative analyses explored how educators experienced the course and applied their learning in practice, including changes to routines, learning environments, documentation, and interactions with children, families, and colleagues. The findings are summarized in Table 2 (below) and presented in full in Supplementary Appendix H. They are organized thematically and mapped according to the Charter’s action areas, with quotations illustrating how educators’ post-course accounts of their learning and practice aligned with the aims of the course and reflect the multiple, interconnected dimensions of health promotion in ECEC. In the discussion, we interpret these findings to explore how educators applied their learning individually and within their centers, with implications for sector-wide improvements regarding health literacy (for educators) and education (for children and their families).

**Table 2:** Educator perspectives of the course and their descriptions of how they applied their learning in practice (themes with selected quotations) mapped to the Charter’s action areas.

Charter action area	Theme from interviews	Representative quotation
1. Building healthy public policy	Supporting centers to align with expectations and needs in their context	“Parents have the perspective that you’re getting a child ready for school, and our governing body has a very play-based approach… and we do our best to instil that [but] it’s very hard sometimes to specify how we’re encouraging literacy… Being able to have resources that specifically link to that literacy learning aspect [to show parents what we are doing], it’s a big thing.” (Jenny)
	Developing policy and practices for health promotion within centers	“We were thinking of updating our centre policy… having links to this program in there [and] incorporated into orientation packs so parents have additional information.” (Davina)
	Supporting centers to meet policy and accreditation requirements	“The [reflective] journal’s good as part of the centre’s [weekly] critical reflection process… as part of our documentation [for accreditation purposes].” (Amy)
2. Creating supportive environments	Supporting educators to facilitate healthy routines for children	“We would do it [during] transitions, especially before lunchtime… since incorporating the books, if a child’s not washing their hands, their friends are dobbing them in now and using that terminology: ‘You’re going to get bad bugs, you’re going to get sick.’” (Davina)
	Supporting educators to facilitate health-promoting environments	“We’ve found them very beneficial… posters already up… it was very helpful to have those two connections straight away. The children… make that connection quite quickly.” (Jenny)
	Ensuring safe and healthy learning environments	“Even [with] the more non-verbal kids… you can use the books to help you, with pictures, to [ask] ‘how are you feeling? Is it something in your belly?’” (Linda)
	Helping children to make real-life connections	“I do think of the germs going down the sink because the kids [have] really jumped on that. So now, when I’m washing my hands, I'm like, oh yeah, germs are going down.” (Jenny)
	Enabling developmentally responsive adaptations	“I hadn’t seen a program [like the books] that really suited the earliest [years]… that was really appealing to me.” (Linda)
3. Strengthening community action	Encouraging knowledge sharing among staff in a center	“These books and the course [were] useful for me as educational leader… to share my [health literacy] knowledge and give tips and advice [to other educators].” (Amy)
	Supporting center–family connections	“We went back to our policy and… sent an e-mail out to ensure that the families and children are washing their hands… we’re going to utilize the resources and add it into the orientation pack.” (Davina)
	Connecting centers through the online course	“[The discussion boards facilitated] a little online community… you’ll find educators always talk to each other… I’m already a member of multiple Facebook groups… so this [health literacy learning] becomes part of our normal conversations.” (Amy)
4. Developing personal skills	Developing the skills of educators	“It’s huge to have that knowledge… it’s not just reading a book, it’s making those connections.” (Jenny)
	Developing the skills of children	“Since using the books… I’ve seen children automatically go and wash their hands… they talk about how they do it at home.” (Amy)
5. Reorienting health services	Educators promoting preventative health practices	“We utilized that book *[When I am sick]* to educate the children on the importance of washing their hands… during COVID everyone was big on hand sanitising… but it slipped. The children now use the terminology from the book in other areas.” (Davina)
	Building confidence around health procedures	“The healthy teeth book… was popular because of our dental visits… it helps those children who have had teeth removed… talk about it.” (Shay)

## Discussion

This study evaluated a short, digitally delivered course designed to strengthen ECEC educators’ health literacy and dialogic reading capability, using the Ottawa Charter for Health Promotion as an analytic framework to interpret the findings, rather than as the object of evaluation. Taken together, the quantitative and qualitative findings indicate that the course was fit for its intended purpose. Statistically significant improvements in educators’ health literacy and dialogic reading knowledge, alongside high levels of reported confidence and perceived usefulness, demonstrate that the course supported educators’ understanding of key concepts and their readiness to apply LAB resources in practice. Qualitative findings further show how this learning translated into everyday pedagogical routines, reflective documentation, and interactions with children, families, and colleagues.

Importantly, these changes did not occur in isolation. Educators’ accounts illustrate how individual learning was enacted through the creation of supportive learning environments, shared practices, and policy-aligned routines within ECEC settings. Interpreting these outcomes through the Charter highlights the interaction of multiple action areas, including developing personal skills, creating supportive environments, and strengthening community action, and highlights the value of the Charter for understanding how health promotion is operationalized in contemporary, digitally mediated early childhood contexts.

This discussion includes selected participant quotations from the full qualitative dataset (Supplementary Appendix H) to illustrate and contextualize the researchers’ analytic interpretations. The first section focuses on the intersection of developing personal skills and creating supportive environments, as educators consistently described these domains as closely linked in their everyday enactment of course learning.

### Developing personal skills within supportive early learning environments

#### Developing personal skills + creating supportive environments

A central finding of this study is the way educators’ developing personal skills were enacted through the creation of supportive, health-promoting learning environments. Educators consistently described how increased understanding of health literacy and dialogic reading shifted their everyday pedagogical practices, enabling them to embed health concepts within routines such as handwashing, mealtimes, and transitions. These qualitative accounts are consistent with the statistically significant improvements observed in both dialogic reading knowledge and overall health literacy scores following course completion. They also align with research evidence that highlights the importance of situating health literacy in authentic, everyday routines (Bánfai-Csonka *et al*. 2022).

Across the qualitative data, educators emphasized the value of dialogic reading, recognizing its pedagogical capacity to strengthen children’s language, pre-literacy skills, and meaning-making capacities (Cliffe and Solvason 2023) while forming rich, relational context for health literacy. The open-ended, visual nature of the LAB books—a deliberate design feature known to support health literacy development (Bánfai-Csonka *et al*. 2022)—supported developmentally responsive adaptations across age groups and abilities. For example, Linda (see Table 2) described how the resources enabled participation by children with limited verbal language, illustrating how educator capability and environmental affordances worked together to support inclusive health literacy learning. This is similar to capability models that emphasize how literacy outcomes emerge through the interaction between individual skills and enabling environments (Oades *et al*. 2021).

Educators also reported that repeated use of shared language and imagery strengthened children’s understanding of health behaviors beyond the immediate learning context. For instance, Amy’s quote (Table 2) suggests that health literacy learning was being transferred across settings. Importantly, these reported changes in practice occurred alongside measured gains in educators’ health literacy, suggesting that observed shifts in routines were underpinned by substantive learning rather than perception alone. These findings align with evidence that routines (Duh-Leong *et al*. 2025), combined with relational pedagogy (Cliffe and Solvason 2023), provide powerful foundations for early health promotion, while extending this literature by demonstrating how targeted PD can support educators to operationalize these principles with confidence (Wallace *et al*. 2025b). While these accounts indicate educators’ perceptions of children’s learning and transfer across contexts, child-level outcomes were not directly measured in this study.

### ECEC as a site of policy enactment, not just policy compliance

#### Building healthy public policy

Educators’ accounts indicated that the course functioned as an important bridge to compliance with ECEC legislation (NQS and EYLF2.0) and, importantly, as a mechanism for policy enactment within everyday ECEC practice. In the Australian context, services are under increasing scrutiny to ensure that daily pedagogical and health practices are visible, well documented, and assessable, particularly in relation to NQS Area 2 (Staton *et al*. 2025). Rather than describing policy as an external requirement imposed on practice, educators spoke about using LAB resources to interpret, prioritize, and make visible health-promoting practices in ways that aligned with regulatory expectations. This was particularly evident in relation to the NQS (ACECQA 2018), where health literacy concepts were often perceived as present but deprioritized. As one educator noted, “I know [personal hygiene is] in NQS Area 2, but it’s also a topic that… can be the bottom of the list of importance” (Davina). Participants described LAB resources as prompting renewed attention to these expectations, particularly during periods of heightened health risk such as winter illness outbreak, when policy requirements become more salient in practice, as also noted in research reporting the efficacy of engaging educational methods for promoting handwashing with young children (Younie *et al*. 2020).

Educators also described how the course supported the translation of abstract policy requirements into concrete, documentable practice. Several participants reported incorporating LAB materials into center policies, orientation packs, and critical reflection processes, enabling health promotion to be embedded within existing governance structures rather than treated as an additional burden. Linda reflected that “having any of that [curriculum and standards work] done for us already makes everything simpler and less time-consuming,” highlighting how system-aligned resources can reduce the cognitive and administrative load associated with policy enactment (Noble *et al*. 2020). Thus, the course supported educators to move beyond minimal compliance toward more intentional, defensible health promotion practice, illustrating how early childhood settings can function as active sites of healthy public policy in action.

### From individual learning to collective action

#### Strengthening community action

While the course was undertaken by individual educators, the qualitative findings demonstrate that its effects often extended beyond individual learning to shape collective practices within and across centers. Educators, like Amy (Table 2), described sharing course content, resources, and pedagogical strategies with colleagues, positioning themselves as informal knowledge brokers within their services, similar to other studies (Cramer *et al*. 2022). This knowledge sharing was not framed as top-down dissemination, but as collegial exchange embedded within everyday professional interactions. Such readiness to share and extend learning was supported by the post-course survey findings, with 95% of participants reporting increased confidence in using LAB resources within their centers.

Importantly, educators also described how health literacy learning became integrated into center–family communication. Participants reported updating policies, sending targeted messages to families, and incorporating LAB materials into orientation processes to reinforce consistent health messages across home and service contexts. These accounts suggest that strengthening community action occurred through formal partnerships, and through routine, relational practices that connected educators, families, and children around shared health goals. Educators who had built health literacy skills through the course could then act as ‘health literacy mediators’ within their centers, sharing knowledge and supporting colleagues to embed health-promoting practices (Spencer *et al*. 2021).

Beyond center-level sharing, the online discussion boards embedded within the course supported forms of community action that extended across services. Educators (e.g. Amy, Table 2) described these spaces as aligning with existing professional norms of online collegiality rather than creating additional demands. This finding highlights the value of leveraging familiar digital practices to foster collective reflection and shared learning, similar to previous research that examined the sense of community created within an online community of practice for early-years educators (Wallace *et al*. 2018), reinforcing the Charter’s emphasis on community action as relational and contextually embedded concept rather than one that is formally structured.

### Reorienting health services: ECEC as a community health touchpoint

#### Key contribution to this Special Issue

Health and education are inextricably linked, and high-quality early-years education and care is a powerful tool to support health (Hahn and Barnett 2023). Although ECEC settings are not considered health services in a traditional sense, educators’ accounts strongly suggest that they functioned as community-based health promotion touchpoints, particularly in relation to preventive health practices and children’s early encounters with health systems. Through LAB resources, educators described becoming more confident in addressing health topics that are often perceived as sensitive or complex, including illness, hygiene, dental care, and medical procedures, for example, as demonstrated by Shay (Table 2) who used the narrative resources to support children to make sense of their individual health experiences.

Across the qualitative data, educators repeatedly described using the books to normalize conversations about health and illness, particularly in the aftermath of COVID-19. Participants noted that while hygiene practices had intensified during the pandemic, they had subsequently “slipped,” and the LAB resources provided a developmentally appropriate way to re-embed preventive practices. These accounts suggest that early-years educators were reorienting their everyday pedagogical roles toward sustained health promotion, aligning with the Charter’s call to embed prevention across sectors rather than confining it to healthcare settings (Thomas *et al*. 2025).

Critically, educators also described role modeling health behaviors alongside children, reinforcing the relational nature of health promotion in early childhood contexts. One participant described bringing her own toothbrush into the room to demonstrate dental care practices, framing this as part of her professional responsibility rather than an additional task. Such examples align with a growing body of research demonstrating that ECEC settings operate as trusted, low-threshold environments in which children and families engage with health concepts and procedures through daily, relational practice, positioning early learning as a vital—yet frequently under-recognized—component of community health infrastructure (Bánfai-Csonka *et al*. 2022, Horm *et al*. 2024).

### Implications for Ottawa Charter-aligned evaluation in digital early-years interventions

#### Meta-level contribution

Taken together, the findings of this study demonstrate the continued relevance of the Charter as a framework for evaluating contemporary, digitally delivered health promotion initiatives in early childhood settings. Rather than aligning neatly with a single action area, the course operated across multiple, interacting domains, illustrating how personal skill development, supportive environments, community action, policy enactment, and service reorientation are mutually reinforcing in practice. Using the Charter as an analytic lens enabled these interconnections to be made visible, moving beyond outcome-focused evaluation toward a more systems-oriented understanding of impact. For PD interventions, this approach supports evaluation that captures both individual learning outcomes and how educator learning is enacted within organizational and system contexts.

The study also contributes to emerging discussions about the role of digital determinants in health promotion, particularly in professional learning contexts (Holly *et al*. 2025). The accessibility and flexibility of the online PD supported participation across diverse settings, while embedded discussion features facilitated collegial learning without imposing additional time burdens. These findings suggest that digitally mediated interventions can meaningfully activate Charter principles when they are pedagogically grounded, contextually aligned, and integrated into existing professional practices.

Finally, this study demonstrates how the Charter can function as a reflective framework for identifying both strengths and gaps in implementation, including structural conditions affecting equity, program sustainability, and scalability. For early-years health promotion, where interventions are often embedded within complex educational and regulatory systems, such evaluative approaches offer a powerful means of understanding how health is produced in everyday settings and how the Charter’s principles can continue to guide health promotion in a rapidly changing landscape.

### Strengths and limitations

This study has several strengths. The mixed methods design enabled an integrated understanding of the outcomes of the course for educators by combining quantitative evidence of improved health literacy and dialogic reading capability with rich qualitative insights into their experiences. Methodological rigor was enhanced through member checking, peer debriefing, and the explicit use of the Charter as an analytic framework, which provided conceptual depth and supported consideration of both individual and environmental influences. Used in this way, the Charter structured interpretation rather than guiding program design, which may have limited the extent to which some Charter action areas were observable within the available data. The digital delivery of the intervention reflects contemporary modes of professional learning in ECEC, enhancing the relevance and potential scalability of the approach. Mapping of the course content (specifically the glossary of terms) to the HLQ-16 data collection tool demonstrates transparency and highlights knowledge growth among participants was attributable to the course.

Several limitations should be acknowledged. The convenience sample was small and biased toward the Western Australian ECEC context. It consisted of volunteers from selected organizations and online recruitment channels, which may have attracted educators already motivated to engage in health-promoting practice. As such, findings may not be fully generalizable across the broader ECEC workforce (Salkind 2022). Implementation occurred within varied service contexts, and differences in organizational support, available resources, and educator experience likely influenced course uptake and the use of LAB materials. The study did not measure medium- or long-term child-level impacts, limiting conclusions about sustained outcomes. Finally, although the Charter provided a useful framework for interpreting the intervention, its retrospective application to an existing program may have constrained opportunities to examine other action areas, such as community participation or policy-level change.

### Future directions

Future work could extend this study by exploring sustained or multisession models of professional learning to deepen educator capability and support consistent implementation across diverse ECEC contexts. Embedding health literacy more explicitly into ECEC policy, quality assurance systems, and preservice qualifications may help address structural barriers and enhance sector-wide uptake. Further research is needed to ensure equitable access, including codesigning adaptations with under-resourced, rural, and culturally diverse communities. Longitudinal studies examining child-level outcomes and the durability of practice change would strengthen understanding of the intervention’s long-term impact. Opportunities also exist to broaden Charter-aligned evaluation by investigating the roles of community partnerships, digital tools, and intersectoral collaboration in scaling LAB within whole-service approaches to health promotion. Establishing educator communities of practice or leveraging knowledge brokers may additionally support sustainability and promote the integration of health literacy across early-years learning environments.

## Conclusion

This study demonstrates that a brief, digitally delivered PD course can strengthen early-years educators’ health literacy and confidence to embed health-promoting practices through everyday pedagogical routines. Applying the Charter provided a valuable framework for evaluating how individual capability, learning environments, and system-level conditions interact in early childhood health promotion. The findings highlight the continued relevance of the Charter for contemporary, digitally mediated interventions and underscore the importance of aligning professional learning with policy and accreditation contexts. LAB represents a promising, scalable approach to advancing health literacy in the early years.

## Supplementary Material

daag087_Supplementary_Data

## Data Availability

The data underlying this article cannot be shared publicly due to ethical concerns for the privacy of the individuals who participated. Limited de-identified data may be shared on reasonable request to the corresponding author.
