# Supplementary material for: Applying the Ottawa Charter to evaluate health literacy outcomes of the Little Aussie Bugs course for Australian early childhood educators
Source: Health Promot Int. 2026 Jul 6;41(4):daag087. doi: 10.1093/heapro/daag087 (PMC13394707; doi:10.1093/heapro/daag087)
Supplement: daag087_Supplementary_Data [file daag087_supplementary_data.zip › Supplementary Figure - Appendix B.pdf]

Appendix B: **Reflective journal**.

Little Aussie Bugs

Promoting health literacy at your service!

Reflective Journal

Your name: …………………………………………………………………………………………

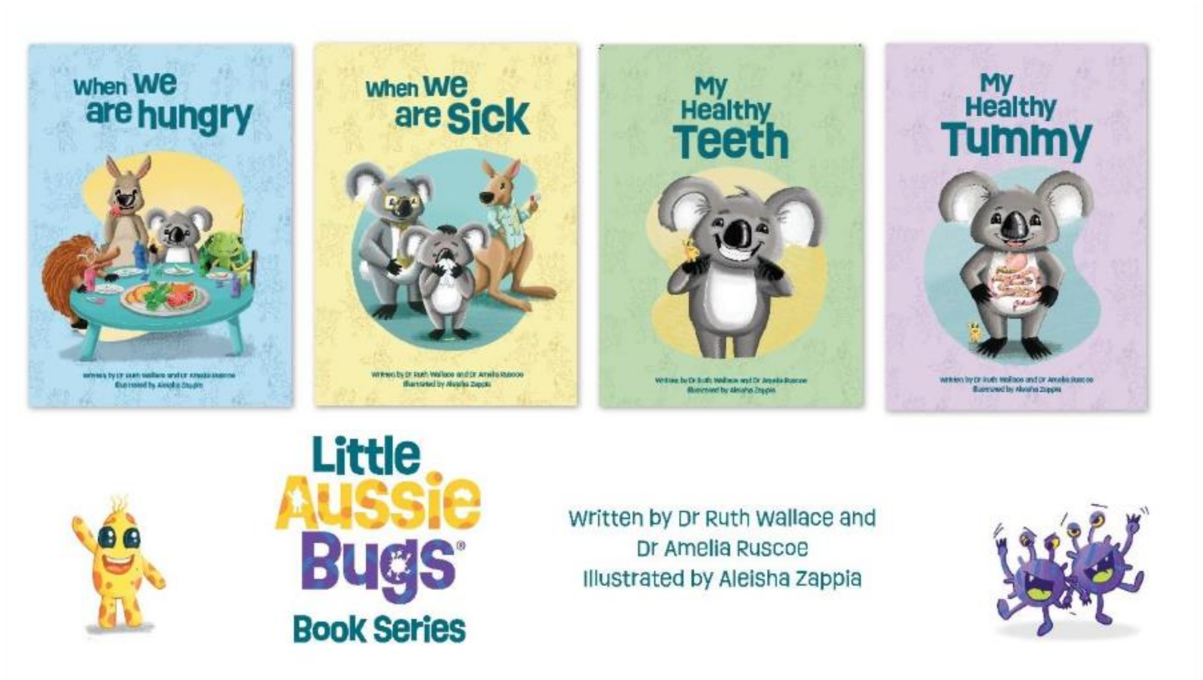

Instructions for use

This reflective journal is designed to be used with the **Little Aussie Bugs** professional development course.

Your **reflective journa**l is a space for you to make notes about what you learn while completing the course and to plan new learning activities for use in your work.

A reflective journal is a powerful tool that helps you:

Learn from your experiences, build on existing knowledge and identify areas for growth and development.

Record your thoughts, feelings and observations. This self-reflection helps you to understand your teaching practices, interactions with children and decision-making processes.

Explore any challenges you may experience, celebrate your successes and identify areas for professional growth.

Continually improve your practice by connecting new ideas with existing knowledge, adjusting your teaching strategies and making plans for new ways of doing things.

Course learning outcomes

On completion of this course, participants will have:

Built their capacity and confidence to engage children and their families with health literacy messages in early learning settings.

Developed the skills to ensure inclusive and accessible health literacy messaging is embedded at Early Learning settings.

Introduction to health literacy

Element 2.1.3: Healthy eating and physical activity are promoted and appropriate for each child.
EYLF Outcome 5.2 : Children engage with a range of texts and gain meaning from these texts.
EYLF Outcome 3.3: Children take increasing responsibility for their own health and physical wellbeing.

Write a couple of sentences explaining **health literacy** to yourself.

Why do you think it is important to teach children about **health literacy**?

Health literacy in action

Element 2.1.3: Healthy eating and physical activity are promoted and appropriate for each child.
EYLF Outcome 5.2: Children engage with a range of texts and gain meaning from these texts.

Write some **health literacy** examples that you remember from watching the video.

Do they remind you of some health literacy examples of your own or at your service?

Lifelong literacy and health foundations

Element 2.1.3: Healthy eating and physical activity are promoted and appropriate for each child.
EYLF Outcome 5.2: Children engage with a range of texts and gain meaning from these texts.

Think about how you currently read books with children. How do you support children's pre-literacy skills during reading time?

Is there anything new that you would like to try? Jot down your ideas and make a plan to try them.

Dialogic reading

Element 2.1.3: Healthy eating and physical activity are promoted and appropriate for each child.
EYLF Outcome 5.2: Children engage with a range of texts and gain meaning from these texts.

In your own words, describe dialogic reading to yourself.

What should you remember to do while using this strategy with children?

Building common language

Element 2.1.3: Healthy eating and physical activity are promoted and appropriate for each child.
EYLF Outcome 5.2: Children engage with a range of texts and gain meaning from these texts.

What health routines and rituals do you use at your service?

How will you use the **Little Aussie Bugs** books to build a common language in your centre?

When we are hungry

Element 2.1.3: Healthy eating and physical activity are promoted and appropriate for each child.
EYLF Outcome 5.2: Children engage with a range of texts and gain meaning from these texts.

Write down the activities that you could run with children, using the activity ideas from the book.

Add your own ideas for learning activities.

Note *why* you think these learning activities are important, i.e., what **health behaviour** are you focusing on?

Plan - when, where and how - you will use these learning activities at your service?

When we are sick

Element 2.1.3: Healthy eating and physical activity are promoted and appropriate for each child.
EYLF Outcome 5.2: Children engage with a range of texts and gain meaning from these texts.

Write down the activities that you could run with children, using the activity ideas from the book.

Add your own ideas for learning activities.

Note *why* you think these learning activities are important, i.e., what **health behaviour** are you focusing on?

Plan - when, where and how - you will use these learning activities at your service?

My healthy teeth

Element 2.1.3: Healthy eating and physical activity are promoted and appropriate for each child.
EYLF Outcome 5.2: Children engage with a range of texts and gain meaning from these texts.

Write down the activities that you could run with children, using the activity ideas from the book.

Add your own ideas for learning activities.

Note *why* you think these learning activities are important, i.e., what **health behaviour** are you focusing on?

Plan - when, where and how - you will use these learning activities at your service?

My healthy tummy

Element 2.1.3: Healthy eating and physical activity are promoted and appropriate for each child.
EYLF Outcome 5.2: Children engage with a range of texts and gain meaning from these texts.

Write down the activities that you could run with children, using the activity ideas from the book.

Add your own ideas for learning activities.

Note *why* you think these learning activities are important, i.e., what **health behaviour** are you focusing on?

Plan - when, where and how - you will use these learning activities at your service?

Using the books at your service

Take a few moments to reflect on what you have learned about health literacy and dialogic reading by completing this course, and how you are going to implement the books at your service:

What did you learn?
about health literacy, dialogic reading...or anything else?
what was your initial reaction? What was the reaction of others?
what was good or challenging about completing the course?

So what?
what did this teach me about myself?
what did this teach me about my knowledge or skills?
what could I have done differently?

Now what?
what do I need to do anything to improve my knowledge or skills?
how am I going to share my new knowledge with others?
what are your plans for implementing some of the activities at your service?

Useful resources

Module 2 Health Literacy in early learning

Health literacy	Australia's health/ health literacy, AIHW, 2022<br>Defining health literacy: World Health Organization, 2024<br>How to teach health literacy

Module 3 Dialogic reading

Life-long literacy and health	EYLF 2.0: Belonging, being & becoming<br>Raising Children Network
Dialogic reading	Blog - What is dialogic reading?<br>Dialogic reading for 2- and 3-year-olds<br>Serve and return

Module 4 Introducing the Little Aussie Bugs

When we are hungry (Quiz)	Hunger signs<br>Eat a Rainbow<br>Vegetable nutrition<br>Eating family-style meals
When we are sick (Quiz)	Personal Hygiene for children (Health Direct)<br>Looking after sick children<br>Boosting your child's immune system
My healthy teeth (Quiz)	Factsheets - Children's oral health<br>Dairy and dental health
My healthy tummy (Quiz)	Growing healthy toddlers - Hunger signs<br>Helping fussy eaters
