# Supplementary material for: Applying the Ottawa Charter to evaluate health literacy outcomes of the Little Aussie Bugs course for Australian early childhood educators
Source: Health Promot Int. 2026 Jul 6;41(4):daag087. doi: 10.1093/heapro/daag087 (PMC13394707; doi:10.1093/heapro/daag087)
Supplement: daag087_Supplementary_Data [file daag087_supplementary_data.zip › Supplementary Figure - Appendix G.pdf]

Appendix G: **Full quantitative analyses and results**.

Analyses tested for differences before and after undertaking the course in participants' responses to:

1. **Questions about dialogic reading**.
    a. Response (correct/incorrect) to each question individually.
    b. Total correct answers across the four questions.

2. **Questions about health literacy**.
    a. Response to each question individually.
    b. For an overall 'health literacy score'.

All analyses were run in R (version 4.4.1) within R Studio (2025.09.2 Build 418).

1. **Dialogic reading (DR)**
    a. Responses to individual DR questions.

Logistic regression models were used to test whether the probability of answering each DR question correctly changed pre and post course. 'Time' (i.e., pre or post) was included as a fixed effect and individual ID was included as a random effect. These models were run using the glmer function in the 'lme4' (v1.1-36) R package.

Table 1 shows the number of incorrect and correct answers to each question pre and post course. The Estimate gives the change post-course relative to pre-course on the log odds scale. These are all positive, indicating higher probability of correct answers post-course, although the difference was only significant ($p < 0.05$) for question 1.

Table 1. Summary of analyses comparing responses (incorrect/correct) to individual DR questions pre and post course.

**DR question**	**Pre**		**Post**		**Estimate**	**Std. Error**	**z**	**p**
	**No. incorrect**	**No. correct**	**No. incorrect**	**No. correct**				
1. Dialogic reading involves?	12	27	5	34	7.882	2.160	3.650	0.0003
2. Which is NOT a literacy goal of Dialogic reading?	12	27	6	33	1.038	0.635	1.634	0.102
3. Dialogic Reading is an appropriate strategy because?	4	35	3	36	0.888	1.397	0.636	0.525
4. Which is NOT a pedagogical benefit of Dialogic reading?	23	16	21	18	0.264	0.514	0.513	0.608

b. Total correct answers across the four DR questions.

At each timepoint, participants' total score on the dialogic reading questions was calculated by adding up the number of correct responses (i.e., scores between 0 and 4). The difference between scores pre and post course was analysed using an ordinal logistic regression (OLR) model with 'Time' (i.e., pre or post) as the predictor variable and individual ID included as a random effect, run using the clmm function in the 'ordinal' (v2023.23-4.1) R package. An

ordinal logistic regression model was used because the number of different possible scores is low, so it isn't appropriate to treat them as continuous.

Table 2 shows the mean scores pre- and post-course, and the summary of the OLR model. The 'Estimate' is an estimate of the change in score post-course compared with pre-course, noting that for an OLR model this is on the log odds scale. Total scores improved significantly post-course, compared with pre-course (p = 0.045).

Table 2. Summary of analysis comparing total scores on DR questions pre- and post-course.

Mean_score_pre	Mean_score_post	Estimate	Std. Error	z	p
2.692	3.103	1.714	0.854	2.007	0.045

2. **Health literacy**
a. Response to individual health literacy questions.

The frequencies of responses to each of the health questions pre and post course are shown in Figure 1, ranging from 'very easy' to 'very difficult'. See Table 3 for full details of the question relating to each number. For all questions, more people answered 'very easy' or 'easy' (yellow and green bars) post-course, suggesting improving health literacy.

Figure 1. Plot summarising frequency of each response to each of the health literacy questions pre- and post-course.

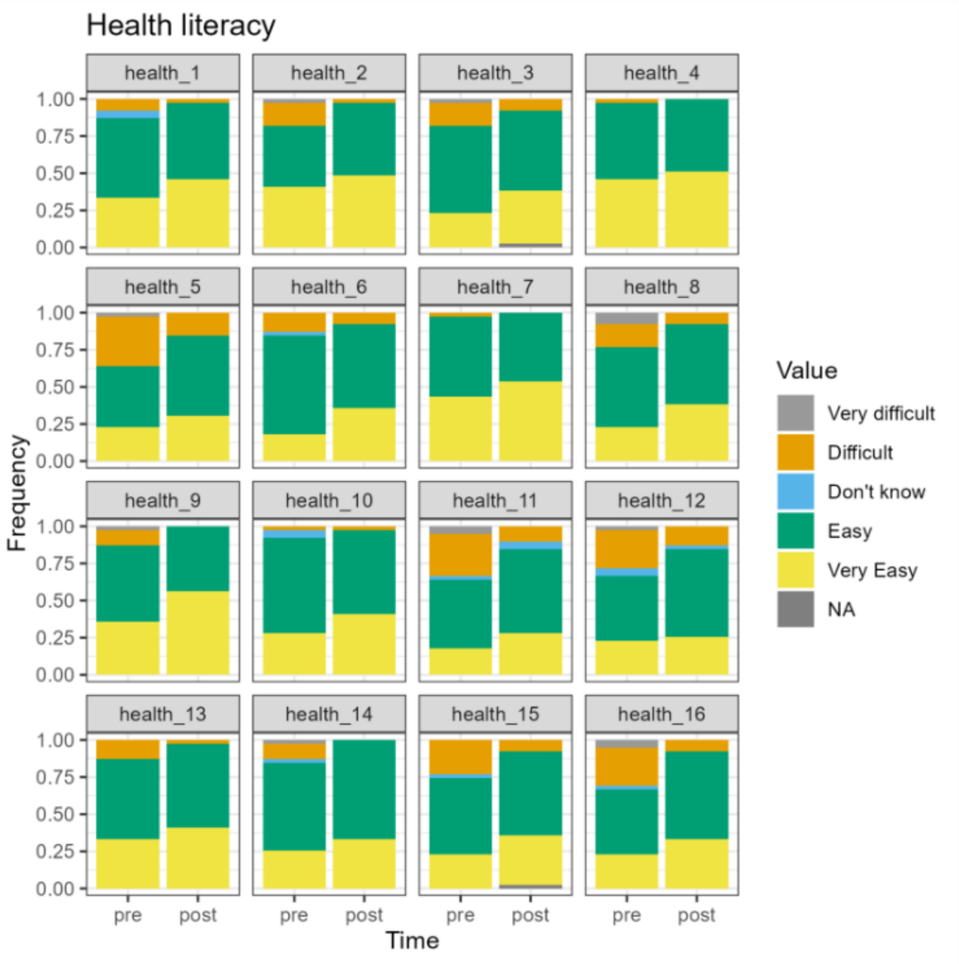

Ordinal logistic regression (OLR) models were run for each question, again with ‘Time’ (i.e., pre or post) as the fixed predictor variable and individual ID included as a random effect, run using the clmm function in the ‘ordinal’ (v2023.23-4.1) R package. These are summarised in Table 3. The responses were ordered as ‘very difficult’, ‘difficult’, ‘don’t know’, ‘easy’, ‘very easy’. Therefore, positive values in the ‘Estimate’ column correspond to a shift in responses towards the easy end post-course compared with pre-course (i.e., improving health literacy). These values were positive for all questions. Those where the difference was significant ($p < 0.05$) are shown in bold italics.

Table 3. Summary of analyses comparing responses to individual health literacy questions pre- and post-course.

**Question number**	**Health question**	**Estimate**	**Std. Error**	**z**	**p**
health_1	***Find information about treatments for illnesses that concern you?***	***1.465***	***0.739***	***1.983***	***0.047***
health_2	Find out where to get professional help when you are ill?	0.893	0.524	1.704	0.088
health_3	***Understand what your doctor says to you?***	***7.108***	***1.313***	***5.414***	***0.000***
health_4	Understand your doctor's or pharmacists instructions on how to take a prescribed medicine?	0.640	0.672	0.952	0.341
health_5	***Judge when you need to get a second opinion from another doctor?***	***1.398***	***0.575***	***2.432***	***0.015***
health_6	***Use information your doctor gives you to make decisions about your illness?***	***4.715***	***0.002***	***2404.536***	***0.000***
health_7	Follow instructions from your doctor or pharmacist?	0.743	0.590	1.258	0.208
health_8	Find information on how to manage mental health problems such as stress and depression?	1.546	0.608	2.543	***0.011***
health_9	***Understand warnings about behaviour (e.g., smoking, low physical activity, and drinking too much alcohol)?***	***2.127***	***0.774***	***2.747***	***0.006***
health_10	Understand why you need health screenings?	1.119	0.641	1.745	0.081
health_11	***Judge if the health information on health risks in the media is reliable (e.g., from TV or internet)?***	***6.416***	***0.004***	***1786.076***	***0.000***
health_12	Decide how you can protect yourself from illness based on information from the media?	0.933	0.520	1.794	0.073
health_13	Find out about activities that are good for your mental wellbeing?	0.784	0.534	1.467	0.142
health_14	Understand advice on health from your family or friends?	0.869	0.509	1.707	0.088
health_15	***Understand information on the media on how to get healthier?***	***1.138***	***0.537***	***2.121***	***0.034***
health_16	***Judge which everyday behaviour is related to your health?***	***1.773***	***0.610***	***2.903764***	***0.004***

a. Construction and analysis of a composite score for health literacy.

To construct a composite score for health literacy, responses to each of the 16 health questions were coded numerically as follows:

Response	Numeric score
Very difficult	0
Difficult	1
Don't know	2
Easy	3
Very Easy	4

These scores were then summed across the 16 questions for each participant at each timepoint (pre- and post-course). This resulted in a total possible score range of 0 – 64 per participant at each timepoint, with higher values indicating that they typically gave responses toward the 'easier' end of the spectrum (suggesting higher health literacy).

A linear mixed model was run to test whether this overall health literacy score differed pre- and post-course. As with the other analyses outlined above, 'Time' (pre vs post) was included as a fixed predictor variable and individual ID was included as a random effect. The model was run using the lmer function in 'lme4' (v1.1-36), assuming a gaussian distribution. Results of this model, along with mean scores pre- and post-course, are shown in Table 4. Here, the Estimate indicates the raw difference of the post-course score compared with the pre-course score. The mean score was 5.9 points higher post-course than it was pre-course, and this difference was significant ($p = 0.0002$).

Table 4. Summary of analysis comparing total health literacy scores pre- and post-course.

mean_pre	SE_pre	mean_post	SE_post	Estimate	Std. Error	df	t	p
46.462	1.815	52.359	1.314	5.897	1.405	38	4.197	0.0002

This analysis was also run with highest qualification and years of experience included as additional fixed effects. The results of this model are summarised in Table 5. Neither of these factors was significantly associated with participants' total health literacy scores ($p > 0.05$), but Time remained significant even after accounting for these ($p = 0.0002$).

Table 5. ANOVA table summarising results of linear mixed model testing association of Time, Years experience and Highest qualification with total health literacy score.

factor	Sum Sq	Mean Sq	NumDF	DenDF	F value	p.value
Time	678.205	678.205	1	38	17.618	0.0002
Years_experience	49.298	16.433	3	31	0.427	0.735
Highest_qual	304.193	76.048	4	31	1.976	0.123
