# Supplementary material for: Applying the Ottawa Charter to evaluate health literacy outcomes of the Little Aussie Bugs course for Australian early childhood educators
Source: Health Promot Int. 2026 Jul 6;41(4):daag087. doi: 10.1093/heapro/daag087 (PMC13394707; doi:10.1093/heapro/daag087)
Supplement: daag087_Supplementary_Data [file daag087_supplementary_data.zip › Supplementary Text and Tables - Appendices A-H (except B and G).docx]

**Appendix A**: **Summary of the course design, including how the content of each module aligns with the ECEC curriculum**.

| **Course module** | **Content taught** | **Link to ECEC curriculum** |
| --- | --- | --- |
| Health literacy in early learning | Introduction to health literacy  Health literacy in action | NQF Element 2.1.3  EYLF Outcome 5.2  EYLF Outcome 3.3 |
| Life-long literacy and health | Life-long literacy and health foundations  Dialogic reading  Building a common language | NQF Element 2.1.3  EYLF Outcome 5.2 |
| Introducing the Little Aussie Bugs | When we are hungry  When we are sick  My healthy teeth  My healthy tummy | NQF Element 2.1.3  EYLF Outcome 5.2 |
| Bringing it all together | Using the books at your service  Reflection  Share |  |

**Appendix C**: **Pre‑ and post‑course questionnaires**.

Thank you for your participation in this important research. The aim of this study is to co-design, deliver and evaluate online professional development (PD) to accompany the Little Aussie Bugs dialogic book set, and support early years educators to build health literacy among children aged two to four years attending Australian Early Childhood Education and Care (ECEC) services.

You will be asked to complete this survey at the start of the online professional development course, and again at the end. On both occasions, the survey should take no longer than 10 minutes to complete.

Project title: Little Aussie Bugs: Designing, delivering and evaluating online professional development to support early years educators

Approval Number: 2023-04496-WALLACE

Principal Investigator: Ruth Wallace

I have read the (or someone has read it to me in a language that I understand). By providing my consent, I acknowledge that I:

- have been provided with a copy of the Participant Information Letter explaining the research study.
- have read and understood the information provided.
- have been given the opportunity to ask questions and have had questions answered to my satisfaction.
- can contact the research team if I have any additional questions.
- understand that participation in the research project will involve:
  - piloting the online professional development on a learning management system (Canvas) – you will be provided with a set of the Little Aussie Bugs books as part of this process.
  - completing a pre-course and post-course survey (as part of the piloting process).
  - an invitation to an interview with the researcher to explore your perceptions of the books post-course and how you have used these with the children you educate. The interview will be audio recorded, conducted via TEAMS (or similar) and will take approximately 30 to 45 minutes.
- understand that the information provided will be kept confidential, and that my identity will not be disclosed without consent.
- understand that I am free to withdraw from further participation at any time, without explanation or penalty. Any data you have provided (feedback forms and/or an interview) to that point will not be included.
- freely agree to participate in the project.
- understand the data collected may be used only for the purposes of this research project.

I consent  (1)

I do not consent  (2)

*Skip To: End of Survey If Thank you for your participation in this important research. The aim of this study is to co-desig... = I do not consent*

This next set of questions is about you.

Q1. In what state or territory is the ECEC service at which you work?

Australian Capital Territory  (4)

New South Wales  (5)

Northern Territory  (6)

Queensland  (7)

South Australia  (8)

Tasmania  (9)

Victoria  (10)

Western Australia  (11)

 Q2. What is your current role at the centre where you work?

Trainee  (1)

Educator  (2)

Educational leader  (3)

2IC  (4)

Director  (5)

Other  (6) __________________________________________________

Q3. What is your age?

<18 years  (1)

18-25 years  (2)

26-35 years  (3)

36-45 years  (4)

46-55 years  (5)

>55 years  (6)

*Skip To: End of Survey If What is your age? = <18 years*

Q4. With which gender do you identify?

Male  (1)

Female  (2)

Non-binary / third gender  (3)

Prefer not to say  (4)

Q5. What is your highest level of qualification?

Working towards a Certificate III in Childcare Services  (1)

Certificate III in Childcare Services  (2)

Diploma of Childcare Services  (3)

Undergraduate degree  (4)

Other  (5) __________________________________________________

Q6. How long have you worked in the Early Childhood Education and Care sector?

< 1 year  (1)

1-5 years  (2)

6-10 years  (3)

> 10 years  (4)

The next set of questions are about health literacy. Health literacy is defined as "... how people access, understand and use health information in ways that benefit their health". (AIHW, 2023)

Q7. How easy/difficult is it for you to:

|  | Very easy (1) | Easy (2) | Difficult (3) | Very difficult (4) | Don't know (5) |
| --- | --- | --- | --- | --- | --- |
| Find information about treatments for illnesses that concern you? (1) |  |  |  |  |  |
| Find out where to get professional help when you are ill? (2) |  |  |  |  |  |
| Understand what your doctor says to you? (3) |  |  |  |  |  |
| Understand your doctor's or pharmacists instructions on how to take a prescribed medicine? (4) |  |  |  |  |  |
| Judge when you need to get a second opinion from another doctor? (5) |  |  |  |  |  |
| Use information your doctor gives you to make decisions about your illness? (6) |  |  |  |  |  |
| Follow instructions from your doctor or pharmacist? (7) |  |  |  |  |  |
| Find information on how to manage mental health problems such as stress and depression? (8) |  |  |  |  |  |
| Understand warnings about behaviour (e.g., smoking, low physical activity, and drinking too much alcohol)? (9) |  |  |  |  |  |
| Understand why you need health screenings? (10) |  |  |  |  |  |
| Judge if the health information on health risks in the media is reliable (e.g., from TV or internet)? (11) |  |  |  |  |  |
| Decide how you can protect yourself from illness based on information from the media? (12) |  |  |  |  |  |
| Find out about activities that are good for your mental wellbeing? (13) |  |  |  |  |  |
| Understand advice on health from your family or friends? (14) |  |  |  |  |  |
| Understand information on the media on how to get healthier? (15) |  |  |  |  |  |
| Judge which everyday behaviour is related to your health? (16) |  |  |  |  |  |

The next set of questions are about your understanding of dialogic reading.

Q8. Dialogic Reading involves:

a. Reading a book aloud to children on the mat.  (1)

b. Having a general discussion with a group of children about the topics in a book.  (2)

c. Retelling a book in your own words rather than reading the text on the pages.  (3)

d. Walking through a picture book with a child or children using dialog to reach understanding of what is represented on each page.  (4)

Q9. How Dialogic Reading supports literacy development. Dialogic Reading is a core strategy for supporting the literacy development of young children. Which of the following is NOT a literacy goal Dialogic Reading?

a. To expand children’s vocabulary.  (1)

b. To teach children to sound out words to read.  (2)

c. To build children’s comprehension skills.  (3)

d. To build children’s oral language skills.  (4)

Q10. Why Dialogic Reading is appropriate for young children. Dialogic Reading is an appropriate strategy for children 1-3 years because:

a. Children this age require ‘serve and return’ to support vocabulary and comprehend what they view in books.  (1)

b. Children like to be read to when they are young.  (2)

c. Children need a logical explanation of a book from an adult.  (3)

d. It helps them learn to read words before they start school.  (4)

Q11. How Dialogic Reading supports health literacy.  Dialogic Reading is a useful pedagogical strategy to support the development of health literacy. Which of the following is NOT a pedagogical benefit of Dialogic Reading?

a. It provides a dialogic platform for applying new knowledge in other learning experiences  (1)

b. It makes the most of natural adult-child interactions to support learning.  (5)

c. It is fun and learning should be fun.  (3)

d. It encourages retention of key words and phrases that can be applied to everyday routines.  (4)

**Post-course only questions:**

Thank you for completing the online professional development. Please now answer these few questions about the course:

Q12. Please rate the statements below about the Little Aussie Bugs online professional development

|  | Strongly agree (1) | Somewhat agree (2) | Neither agree nor disagree (3) | Somewhat disagree (4) | Strongly disagree (5) |
| --- | --- | --- | --- | --- | --- |
| I found the online professional development engaging (1) |  |  |  |  |  |
| The length of the online professional development was appropriate (2) |  |  |  |  |  |
| The content of the online professional development was easy to understand (3) |  |  |  |  |  |
| The online professional development improved my confidence to use the Little Aussie Bugs resources at the centre (4) |  |  |  |  |  |
| The online professional development improved my knowledge of health literacy (5) |  |  |  |  |  |
| The online professional development improved my knowledge about dialogic reading (6) |  |  |  |  |  |
| The downloadable resources available from the online professional development were useful (7) |  |  |  |  |  |

Q13. Is there anything else you would like to add about the course?

________________________________________________________________

________________________________________________________________

________________________________________________________________

________________________________________________________________

________________________________________________________________

Q14. Would you be prepared to engage with the researchers in an interview once you have used the books with the children at your service? If so, please click on this link to leave your details

LINK TO BE ADDED  (1)

**Appendix D**: **LAB course content (as represented by glossary terms and learning outcomes) mapped to the HLQ-EU-16 Health questions**.

| **Health question** | **LAB course content* (glossary terms** – **link to learning outcomes)** |
| --- | --- |
| Q1: Find information about treatments for illnesses that concern you | Antibiotics, Infection, Immune system, Medical or health professional, Prescription, Thermometer, Preventative health – find and understand information about illness and treatment. |
| Q2: Find out where to get professional help when you are ill | Medical or health professional, Prescription, Preventative health, Oral health, Early Childhood Caries (ECC) – explain types of professionals and referral pathways. |
| Q3: Understand what your doctor says to you | Health literacy, Comprehension, Dialog/dialogue, Multi-modal communication, Medical or health professional –understand health communication. |
| Q4: Understand doctor/pharmacist instructions for medicine | Antibiotics, Prescription, Medical or health professional, Health literacy – understand prescribed medicine and instructions. |
| Q5: Judge when you need a second opinion | Evidence-based strategy, Health literacy, Medical or health professional – think critically and make informed decisions about healthcare advice. |
| Q6: Use information from your doctor to make decisions | Health literacy, Evidence-based strategy, Preventative health, Medical or health professional – interpret and apply professional advice. |
| Q7: Follow doctor or pharmacist instructions | Prescription, Antibiotics, Health literacy, Medical or health professional – adhere to treatment and safe medicine use. |
| Q8: Find information on managing mental health (stress/depression) | Gut health (links physical and mental wellbeing), Self-regulation skills, Empathy, Secure, respectful relationships, Shared belonging, Routines and rituals – understand emotional wellbeing and regulation. |
| Q9: Understand warnings about behaviour (smoking, inactivity, alcohol etc.) | Preventative health, Balanced diet, Australian Dietary Guidelines, Overweight and obesity, Personal hygiene, Hand hygiene – describe behaviours that influence health outcomes. |
| Q10: Understand why health screenings are needed | Preventative health, Thermometer, Oral health, Early Childhood Caries (ECC) – understand screening and early detection concepts embedded in prevention and monitoring. |
| Q11: Judge if media health information is reliable | Evidence-based strategy, Health literacy, references to scientific guidelines (e.g., Australian Dietary Guidelines, AIHW, WHO) – source and scrutinise evidence to find accurate and reliable information. |
| Q12: Decide how to protect yourself from illness based on media info | Preventative health, Hand hygiene, Personal hygiene, Immune system, Infection, Healthy diet / nutrition terms – apply practical prevention strategies. |
| Q13: Find activities good for mental wellbeing | Self-regulation skills, Routines and rituals, Shared belonging, Reciprocal relationships, Empathy, Secure, respectful relationships, Learning activity – support wellbeing through relationships and routine. |
| Q14: Understand advice on health from family or friends | Health literacy, Comprehension, Dialog/dialogue, Reciprocal relationships, Culturally-relevant – interpret and contextualise health advice. |
| Q15: Understand media information on how to get healthier | Australian Dietary Guidelines, Australian Guide to Healthy Eating, Balanced diet, Nutrition, Nutrients, Preventative health, Overweight and obesity – understand foundational health promotion content presented via media. |
| Q16: Judge which everyday behaviour relates to health | Balanced diet, serve size vs. portion size, Personal hygiene, Hand hygiene, Oral health, Tooth decay, Physical activity (via overweight/obesity), Preventative health – connect daily behaviours with health outcomes. |

The glossary of terms (Appendix E) was used to map the course content to the HLQ-16 questions as it is not possible to share the actual online course materials as a supplementary document.

Please note: in addition to the glossary, the following components of the course also align with the HLQ-EU-16 Health questions, but further mapping was not conducted to show this alignment:

- The reflective journal (Appendix B) includes prompts and provocations designed to build health literacy knowledge and skills.
- Engagement in the discussion boards enables participants to share and practice their developing health literacy knowledge and skills.
- Each course module unpacks important health literacy concepts and shows educators how they might use the books at their service.

**Appendix E**: **Glossary of terms provided to course participants**.

LITTLE AUSSIE BUGS: GLOSSARY OF TERMS

**Antibiotics: “***medicines that treat infections and diseases caused by bacteria. Antibiotics do not work against viruses. Penicillin is a well-known antibiotic*” (Health Direct, 2024). They are usually prescribed by a medical doctor, dentist and sometimes nurse practitioners.

**Antioxidants:** These are substances produced by your body or found in foods that play an important role in your health. There are many different types of antioxidants found in fresh fruits and

vegetables, plant oils, nuts and other foods, that protect the health of your body (Health Direct, 2024).

**Appetite**: is defined as “*a person’s desire to eat food*” (Medical News Today, 2024). Appetite can increase and decrease due to a wide range of factors (e.g., stress, being unwell) sometimes causing people to eat more or less than their body needs. Appetite is different to hunger, which is a biological response to a lack of food. Someone could have an appetite for food, even if their body is not showing signs of hunger, and vice versa.

**Australian Dietary Guidelines**: a set of guidelines that “*provide up-to-date advice about the amount and kinds of foods that we need to eat for health and wellbeing. The recommendations are based on scientific evidence, developed after looking at good quality research. By following the dietary patterns recommended in the guidelines, we will get enough of the nutrients essential for good health and also help reduce our risk of chronic health problems such as heart disease, type 2 diabetes, some cancers and obesity*” (Eat for Health, 2024).

**Australian Guide to Healthy Eating**: a food selection guide which visually represents the proportion of the five core food groups recommended for consumption each day by adults and children (Eat For Health, 2024).

**Balanced diet:** or otherwise referred to as a ‘healthy diet’. “*Eating a healthy, balanced diet is an important part of maintaining good health and can help you feel your best. This means eating a wide variety of foods in the right proportions, and consuming the right amount of foods and drinks to achieve and maintain a healthy body weight*” (NHS, 2024). A balanced diet comprises foods from the five core food groups to be consumed every day: vegetables and legumes (beans); fruit; grains and cereals; lean meat, poultry, fish, eggs, legumes (beans), tofu, nuts, seeds; milk, cheese, yoghurt or alternatives (Health Direct, 2024).

**Calcium:** is a mineral essential for building and maintaining healthy bones and teeth throughout life. Almost 99% of the body’s calcium is found in the bones. A small amount of calcium is absorbed into the blood and used for the healthy functioning of the heart, muscles, blood, and nerves. The richest dietary sources of calcium include dairy foods, bony fish (e.g. tinned salmon), and dark green vegetables such as broccoli, rocket and watercress (Healthy Bones Australia, 2024).

**Comprehension:** In the context of using Dialogic Reading, comprehension refers exclusively to oral language comprehension, rather than reading comprehension. Comprehension is reached by

children when they understand what is being communicated by another person. In the early years of

life, oral comprehension typically requires a process of serve and return for the child to complete the feedback loop and check for understanding.

**Constipation:** in relation to children, constipation means they are doing hard poos, having trouble pushing poo out and/or are not pooing regularly. Children can become constipated if they don’t have enough fibre in their diet (fruits and vegetables); drink too much milk and don’t eat enough

solid foods; may have an illness that means they eat and drink less; taking certain medications, e.g., cough medicines. Refer to Health Direct (2024) for more information.

**Culturally relevant**: “*means incorporating awareness, understanding, and responsiveness to the*

*beliefs, values, customs, and institutions (family, religious, etc.) and ethnic heritage of individuals or those identified cultures of persons with specific disabilities (for example hearing impaired), into*

*training, treatment, and services designed to impact upon, or meet the needs of individuals or groups*” (Law Insider, n.d.)

**Detrimental**: means “*causing harm or damage*” (Cambridge Dictionary, 2024). Something can have a detrimental outcome or impact (e.g., certain chemicals can have a detrimental impact/outcome on

the environment) or can be detrimental to (e.g., their decision could be detrimental to the future of the school).

**Dialog/ dialogue:** an oral language exchange (conversation) between two or more people.

**Dialogic Reading:** is a process of engaging with a book where a child and more capable other person engage in dialog about the book rather than ‘reading’ a story. Illustrations, and in some instances, words and phrases on each page are used as prompts for conversation. Conversations may include

simple recognition, naming and labelling of objects, ideas, and feelings, making sense of what is viewed, and linking back to prior knowledge through retelling of real or imagined experiences.

**Dietary fibre:** Dietary fibre is found in the indigestible parts of cereals, fruits and vegetables and helps to keep our digestive systems healthy. Most Australians don’t eat enough fibre, which is linked to conditions such as constipation, irritable bowel syndrome (IBS), diverticulitis, heart disease and some cancers (including bowel) (Better Health Channel, 2024).

**Digestion**: the food and drink we consume must be broken down into smaller molecules of nutrients so that it can be absorbed into the blood and carried to cells throughout our body. **Digestion** is the process which breaks down food and drink so that the body can use the nutrients they carry to provide energy and other important functions (Children’s Wisconsin, 2024).

**Early Childhood Caries (ECC):** is tooth decay that affects children aged five years or younger. It affects the baby (deciduous) teeth, causing pain, eating difficulties, development and sleep

problems, time off school and it increases the risk of tooth decay in the adult teeth (Andrew, 2021). ECC is preventable by introducing good dental hygiene habits (brushing and flossing), good feeding practices (limiting the use of bottle and dummies) and good dietary habits (limiting sugary foods and drinks) early in life (Better Health Channel, 2024).

**Empathy:** the ability to understand and share the feelings of another person.

**Evidence-based strategy:** a strategy supported by published research, professional expertise, and

specific knowledges relevant to a particular group or context (for example, early learning) known to improve the quality and efficiency of professional practice.

**EYLF 2.0:** The Early Years Learning Framework for Australia is the Australian Children’s Education and Care Quality Authority [ACECQA] approved learning framework mandated by the federal

government for birth-8 in Australia, updated from its original 2009 version in 2022 (2.0). The five learning outcomes are the curriculum for Birth-5 years. The Principles and Practices are quality

assessed in accordance with the National Quality Standards for children Birth-8 years.

**Gut health**: the gut is your gastrointestinal system, which includes your stomach, small and large intestine. The health of your gut can influence your physical and mental wellbeing and how you feel from day to day. There are trillions of microorganisms living in your gut (gut microbiome) that are essential for healthy gut function. Symptoms of poor gut health can include bloating, sore tummy, flatulence, nausea, constipation or diarrhoea (Health Direct, 2024).

**Hand hygiene:** a general term referring any action of hand cleaning, including: washing hands with the use of water and soap or applying an alcohol-based handrub (liquids, gels or foams) to the surface of the hands. When performed correctly, hand hygiene works to reduce the number of microorganisms on the hands and is the single most effective action to protect you from infections or preventing you from spreading these infections to others (Hand Hygiene Australia, 2009).

**Health literacy:** is defined by the World Health Organization (WHO) as referring broadly to “*the ability of individuals to gain access to, understand and use information in ways which promote and*

*maintain good health for themselves, their families and their communities” (WHO, 2024).* Read more about health literacy by following the WHO link.

**Immune system**: its job is to “*protect the body from infection. It recognises ‘invaders’ such as*

*bacteria, viruses and fungi and abnormal cells like cancer cells. It creates an immune response and helps the body fight the invasion*” (Health Direct, 2024). Read more here

**Infection:** defined as “*the invasion and growth of germs in the body. The germs may be bacteria, viruses, yeast, fungi, or other microorganisms. Infections can begin anywhere in the body and may spread all through it. An infection can cause fever and other health problems, depending on where it occurs in the body. When the body’s immune system is strong, it can often fight the germs and cure an infection.*” (NCI, n.d.)

**Lactose:** is a sugar that naturally occurs in human and animal milk, cheese, and yoghurt. Even though it is a sugar, it is the least cariogenic (i.e., least harmful) to human teeth. Some people have an intolerance to lactose, whereby they may experience symptoms of abdominal pain and bloating.

Read more at the Better Health Channel (2024).

**Learning activity:** any experience that promotes learning. In early learning settings, the evidence is conclusive that in birth-5 settings, the most effective context for learning is play.

**Medical or health professional**: these terms are often used interchangeably and can cover a wide range of specially trained people who can assist you with your health. These can range from medical doctors, nurses, dentists, and other specialists, through to allied health professionals, such as dietitians, physiotherapists, speech therapists and occupational therapists. For many people, their first point of contact will be their General Practitioner (GP) who will often provide a referral to

another medical or health professional. Read more here

**Multi-modal communication:** A combination of communication methods used to communicate. Young children rely upon several means of communicating beyond words, including vocalisations (including crying and sound effects), early utterances including holophrases (single words expressed with different tones to communicate different ideas) and telegraphic speech (2–3-word utterances),

facial expressions, gestures such as pointing, drawing and other mark-making, use of physical objects to demonstrate meaning, and enactment of events using the body.

**Neuroscience:** in the context of early learning and literacy learning, neuroscience refers to scientific knowledge of the brains structure and function, and how this is influenced by sensory input such as those influenced by the pedagogical decisions of educators.

**Nutrients:** “*the substances in food that our bodies process to enable it to function. Your nutrient requirements are influenced by factors including your age, growth stage and activity.*

**Nutrition:** “*the process of taking in and using food; and the scientific study if this*”. Also

“*the substances that you take into your body as food and the way that they influence your health*”. (Cambridge Dictionary, 2024)

**Oral health:** refers to “*the condition of a person’s teeth and gums, as well as the health of the*

*muscles and bones in their mouth. Poor oral health – mainly tooth decay, gum disease and tooth loss*

*- affects many Australian adults and children* (AIHW, 2023).

**Oral language:** language expressed orally through vocal production (as district from written language).

**Overweight and obesity**: “*Overweight and obesity refers to excess body weight. People who are overweight or obese have higher rates of death and illness than people of healthy weight, particularly from cardiovascular disease, type 2 diabetes, some musculoskeletal conditions, and some cancers. The main factors influencing overweight and obesity are poor diet and inadequate physical activity. Increased energy intake from the diet without an increase in energy expenditure through physical activity will result in energy storage as fat and weight gain*” (AIHW, 2023). There are many other factors that can contribute to overweight and obesity, including genetics (e.g., metabolism, appetite, body fat distribution); health inequalities (e.g., living in poverty); environmental factors (e.g., the availability of fast food outlets); societal factors (e.g., media advertising) (AIHW, 2023).

**Personal hygiene:** good personal hygiene helps protect you from infections such as gastroenteritis and COVID-19 and helps to prevent you spreading these infections to other people. Personal hygiene includes: cleaning your body every day; washing your hands with soap and water after going to the toilet; brushing and flossing your teeth; covering your mouth and nose with a tissue (or your sleeve) when sneezing or coughing; washing your hands after handling pets and other animals (Health Direct, 2024).

**Plaque:** a sticky, colourless film of bacteria that forms on teeth. It develops when foods containing carbohydrates (sugars and starches) are frequently left on the teeth. The bacteria that live in our mouths feed on these foods, producing acids, which over a period of time, destroy tooth enamel which causes tooth decay (WebMD, 2023).

**Pre-literacy skills:** Essential skills and knowledge that are learned prior to formally learning how to apply the alphabetic principle to decode words to read or encode words to write at school. Pre-

literacy skills include oral language skills for communication, listening skills, oral vocabulary, book-based vocabulary (that is not commonly used in oral conversations), print and text knowledge, phonological awareness (ability to hear individual words in a sentence, to break down longer words into segments [typically syllables], rhyme, alliteration, ability to discriminate between sounds, identification of individual sounds in words), alphabet knowledge (recognise and name alphabet

letters [graphemes] and the most common sounds [phoneme] they represent, self-regulation skills to sustain focus and persevere with challenging tasks, growth mindset.

**Prescription**: “*a legal document that health practitioners (such as a GP) write for a pharmacist to dispense a specific medicine (e.g., antibiotics). You can’t legally obtain prescription-only medicines without that document” (*Department of Health and Aged Care, 2024).

**Preventative health**: sometimes also known as preventive health or just prevention, means “*any action taken to keep people healthy and well, and prevent or avoid risk of poor health, illness, injury and early death*” (The Australian Prevention Partnership Centre, 2024). This could be something like encouraging people to stop smoking to reduce their risk of developing cancer or encouraging hand washing to prevent the spread of infectious diseases, such as COVID-19. Read more about this at the Sax Institute.

**Principles and practices:** Guiding ideas and ways of educating described in the mandated EYLF 2.0. The Principles and Practices of the EYLF 2.0 are the evidence-based guidelines used to support educators to reflect and refine early childhood education and care in Australia.

**Print and text knowledge:** also known as ‘concepts of print’. The knowledge of the features of a book, including vocabulary for labelling a letter, word, sentence and punctuation, the understanding that text holds meaning, knowledge of directionality (tracking left to right with a return sweep), knowledge of parts of a book and how to navigate a text, awareness of authorship and the role of

the illustrator in communicating complementary ideas. Print and text knowledge is pre-literate knowledge suitable for teaching in pre-school settings that does not require a child to be able to decode and read words independently.

**Reciprocal relationships:** are relationships where two or more people contribute in ways that influence and benefit one another and are responsive to the needs or wants of all involved. In early

learning, reciprocal relationships exist between children, their family, and educations to achieve the best learning and care outcomes for all children.

**Regulate:** in the context of health and the human body, regulate means to “*control or maintain the rate or speed of (a machine or process) so that it operates properly. For example, "a hormone which regulates metabolism*" (Oxford Dictionary, 2024).

**Routines and rituals:** routines are repeated practices that use used systematically to achieve a goal (e.g. the routine of eating fruit together in the mornings). A ritual is a practice that is developed by a particular group of people and builds a sense of belonging (e.g. singing a ‘get ready to eat’ song).

Rituals are unique and diverse and context specific, having been adopted through a shared playful or joyful experience leading to its repetition. Rituals may accompany a routine or be used

spontaneously where appropriate.

**Satiety**: is the “*satisfied sense of feeling full after eating*” (Phillips, 2023). It is important to trust a child to use their in-built sense of satiety at mealtimes, that is, their “*sensitivity to their internal sensations of hunger, appetite and satiety*” (Satter, 2024).

**Secure, respectful relationships:** Children require at least one familiar attachment with an adult in all situations to feel secure that their needs will be met. Relationships with children must be respectful to ensure healthy social and moral development and to reinforce positive personal goals during the development of self-regulation skills. Secure, respectful, and reciprocal relationships is one of the five core principles of the EYLF 2.0.

**Self-regulations skills:** the skills used to ‘regulate’ or keep behaviour ‘in check’. It requires awareness of one’s own thoughts, feelings, and behaviours so that adjustments can be made to assist personal well-being and the well-being of others. Self-regulation is required to build healthy relationships and to be able to maintain a healthy and effective disposition for learning and contributing positively to society.

**Serve and return:** The process of communication exchange, whereby communication skills are reinforced and refined through repeated ‘serve’ (utterance or other multi-modal communication) and ‘return’ (response from other person providing feedback about what has been understood).

Serve and return is a critical process in early childhood required for healthy neural development and the acquisition of speech, language and communication required for lifelong learning and literacy development.

**Serve size vs portion size:** Serve size and portion size are not the same. For each core food group, the Australian Dietary Guidelines identifies the **serve size** of different foods that have roughly the same amount of key nutrients and energy. For example, one slice of bread or one piece of whole fruit. A **portion size**, however, is the actual amount you eat, which could be guided by many factors, including the information manufacturers print on packaging and how hungry you feel.

**Shared belonging:** a sense of belonging to a group, often reinforced through familiar routines and rituals, and through shared experiences over time.

**Thermometer:** is a tool used to measure body temperature, which can indicate fever, a symptom of sickness, or infection. This article from Health Direct provides information about the types of thermometer available, when and how to use one, and what to do if a child’s temperature is higher than normal.

**Tooth decay**: is caused by a build-up of **plaque** on the teeth which can lead to cavities (holes) forming in the teeth, leading to pain and discomfort. Tooth decay can affect people of all ages, including young children with baby teeth. It is strongly related to what you eat and drink (for example sugary foods and drinks) and can be prevented by daily brushing and flossing, eating a healthy diet and regular visits to the dentist (Health Direct, 2024).

**Vitamins and minerals**: are nutrients needed by your body to ensure it works properly. Your body makes energy from the food you eat. Vitamins and minerals are needed to help you use the energy that comes from the food you eat, for strong bones and to help your body fight infections. Only very small amounts of vitamins and minerals are needed in your diet and most people can get all they need from a healthy diet (Health Direct, 2024).

**Appendix F**: **Structured interview guide**.

**Research Aim and Questions**

The **aim**of this study is to co-design, deliver and evaluate online professional development (PD) to accompany the Little Aussie Bugs dialogic book set, and support early years educators to deliver health literacy messages to children aged two to four years attending Australian Early Childhood Education & Care (ECEC).

**Research Questions:**

RQ1: Has completing the online PD affected educators’ levels of health literacy, specific to young children and the ECEC environment?

RQ2: Has completing the online PD affected educators’ understanding of dialogic reading?

RQ3: Has completing the online PD affected educators’ confidence to use dialogic reading resources to deliver health literacy messages to the children they educate?

RQ4: What are educators’ experiences of using these dialogic reading resources to children they educate?

Remind participants that they can ask questions, withdraw, participation is voluntary etc

Interview via TEAMS – read out consent statement and capture on recording

Interview in person – ask participant to sign consent form

Have the books with you

**Interview Questions**

**Please tell me about yourself**

- Age
- how long they have been an educator at this centre
- where is the centre (postcode)
- how long have they been an educator overall
- what qualification they have

**Online professional development**

- How long after completing the online professional learning did you use the books at your service?
- Had you used the resources previously, i.e., before completing the online professional learning

**If YES**: to what extent/how did the online professional learning help you:

- use the resources
- understand how to engage children with dialogic reading

**IF NO:**how did the online professional learning help you to:

- understand how to use the resources (books and educator activities)
- understand how to engage children with dialogic reading

**Features of the online professional learning**

- The journal and reflective activities – how useful were these? How did they help you understand health literacy and how to use the resources?
- The discussion boards - how useful were these? How did they help you understand health literacy and how to use the resources?
- The quizzes - how useful were these? How did they help you understand health literacy and how to use the resources?
- The videos - how useful were these? How did they help you understand health literacy and how to use the resources?

Anything else you want to add about the online professional learning??

**Could you explain why you were interested in the Little Aussie Bugs? (accessing the course/using the books)**

- How aware were you of the importance of promoting personal hygiene, healthy eating and oral health in young children before you used the materials?
- Please describe what you learned from the resources [that you didn’t know already]?
- How did the books change the way you think about your role as an educator [in promoting health literacy]?
- How did the books change the way you think about your own personal hygiene, eating habits and oral health?

What changes did you make or recommend in your centre after using the books and/or activities?

**How did you use the Little Aussie books and educator activities after completing the course?**

- **E.g.,**transitioning from play time to mealtime; specific planned learning activities
- Could you tell me the ways that you used the additional materials (i.e., the educator activities in the back of the book) provided?
- Were they (the educator activities) easy/difficult to use? What would support your use of these activities?

**Were there topics that you would have liked to see included that weren’t?**

• Would you be able to describe any of those for me?

• Where there any parts [of the books] that you didn’t like or find useful?

• What kind of things [in the centre – prompts like toys, etc] helped you to use these resources?

**How do you think the children engaged with the books?**

- Which characters were the children particularly drawn to? Please tell me about this
- Which stories particularly resonated with the children? Please tell me about this
- What examples have you seen where children have engaged in activities covered in the books, e.g., swing that trunk; hand washing; using tongs etc
- What did the children add to the stories?
- Another character?
- Another way of interpreting the stories?

**Any final thoughts?**

Based on your experiences with the online PD and the Little Aussie Bugs book set, what other ideas or approaches do you think could be effective in promoting health literacy in ECEC settings?

Are there any specific topics, formats, or resources that you would like to see developed in the future to support educators in delivering health literacy messages to young children?

**Appendix H**: **Full qualitative findings: Educator perspectives of the course and their descriptions of how they applied their learning in practice (themes with quotations) mapped to the Charter’s action areas**.

| **Ottawa Charter strategic action area** | **Analytic theme from interviews** | **Representative quotations** |
| --- | --- | --- |
| 1. Building health public policy | Supporting centres to align with expectations and needs in their context | … I know [personal hygiene is] in the National Quality Standards for Area 2 [NQS 2], but it’s also a topic that… can be the bottom of the list of importance. So, it was a good reminder [of] how important [personal hygiene] is, especially with winter and illnesses going around. (Davina)    Parents have the perspective that you’re getting a child ready for school, and our governing body has a very play-based approach… and we do our best to instil that [but] it’s very hard sometimes to specify *how* we’re encouraging literacy… to the depths that this was, with a book or with words around the room and things like that. Being able to have resources that specifically link to that literacy learning aspect [to show parents what we are doing], it’s a big thing. (Jenny)    We both wanted to bring it into the centre because it’s a local thing. [The centre] has a lot of local stuff. So, the Australian animal resources…[the] Australian books makes it a bit easier to relate to the kids. Sometimes it’s really hard when you get [international] resources [and] they go ‘what does that mean?’ and you have to try and explain it and then you feel like it loses the meaning. (Shay) |
|  | Developing policy and practices for health promotion within centres | [Following an outbreak of worms] we were thinking of updating our centre policy… having links to this programme in there [and] incorporated into orientation packs so parents have additional information.” (Davina)    [Our service owner/manager] liked the idea of using [the books] to reinforce the practices [around personal hygiene] that we already have. (Shay) |
|  | Supporting centres to meet policy and accreditation requirements | The [reflective] journal’s good as part of the centre’s [weekly] critical reflection process… as part of our documentation [for accreditation purposes]. (Amy)    *When asked what interested them to use the books:*  I just saw a post on Facebook about the course and, being at a new site and… we were expecting to be assessed [by the Education Standards Board], we knew were on the list. So we had been thinking about areas we need to really focus on with our kids, and health and nutrition [is part of that], so we were [already] looking for some sort of programme that we could use. (Linda)    having looked at the [national quality standards and national quality framework], we were going through the areas and realised that having a programme around health and nutrition [would be a good addition]. We do a fair bit around sickness and germs and things like that, but not a lot around the health and nutrition side. And, even in my 20 years, I don’t think there’s really been a good programme that I've ever come across. I just thought, it’s got the four books in there, it covers all of it, this looks like an awesome way to like include it. (Linda)    As part of programming, we have to then link it all to the ELF and Standards even though, in South Australia, we use the literacy and numeracy indicators. So yeah, having any of that [work] done for us already makes everything simpler and less time [consuming]. I'm not sure about the other states [but] we also use the child protection curriculum here in South Australia, and you can link all of that around health and safety... So I guess some of that stuff could be good [to link to when using the books], about body safety, because you are talking about doctors and health and stuff that, you know, it’s OK for a doctor to ask to see under your clothes. (Linda) |
|  | | |
| 2. Creating supportive environments | Supporting educators to facilitate healthy routines for children | I'm putting more of a focus on these [health topics] and reaffirming to everyone the importance of it… obviously sometimes we get busy [but] it’s important to remember how important these things are, especially for making sure, after the children eat, that they have water to wash out their gums and things like that. And, for the preschoolers especially, if they’re feeling restless, it might be a little bit more difficult to get them to brush their teeth. [So], having ways to make it fun, is useful. (Amy)  We encourage the children [by] ask[ing] them a lot of questions [when reading the books] and… at the end of the story, would ask them what did they learn about? What can we do, if we read this before lunch? What are we going to do shortly? We’re going to brush our teeth [or] we’re going to wash our hands. And then, after that, we’ll brush our teeth [or wash our hands]. (Amy)  One of the main factors that we try to teach the children is the importance of washing their hands, but the actual procedure [is difficult for some children to master]. Some children wash their hands too quick[ly]. So, that’s where we teach them to sing like happy birthday [or use] counting. That’s where I found this programme beneficial. (Davina)  We would do it [during] transitions, especially before lunchtime, and ensuring the importance of washing your hands. We also did extensions of science experiments with pepper and water to show [what happens] if you don’t wash your hands. So we tried to do other hands‑on learning opportunities for them to learn about the importance of hygiene. We’ve just started in big groups at the moment, but the science experiment we did in smaller groups settings just so you have that more one‑on‑one interaction with the children. It’s been a good reminder because we’ve noticed, since incorporating the books, if a child’s not washing their hands, their friends are dobbing them in now and using that terminology: ‘You’re going to get bad bugs, you’re going to get sick.’ (Davina)    I think that I have been more into it because the children have been… So, for example, during the morning discussion time, they have been asking me, did you have your fruits today? Did you eat your veggies today? So, it is something that they also reminded me about because they are more aware of it. (Pam) |
|  | Supporting educators to facilitate health-promoting environments | We’ve found them very beneficial, very smooth [for] moving through the process of things. Like the dental book, and now when they’re not feeling well and things like that, it’s been very easy to follow. We’ve found they’ve stayed quite engaged. And we’ve been able to back that up, which has been really helpful, with… the posters already up before reading the book. They’d seen that. I’d put the posters up and we’re on it. So, it was very helpful to have those two connections straight away. [And they] had that real life perspective. They’re aware that, in the bathroom, the hand-washing poster was there, for example, and we’re able to do that. We also had used the ‘my healthy tummy’ book and ‘when we are hungry’ to refresh our eating area… we’re not a catered centre. (Jenny)    [At our lunchbox centre], we’ve created a display with the different resources above where [the children] have their drink bottles and their plates and cutlery if they need it for their own food… It was really easy to have them engaged with the resources as well as the books. [They] are really able to make that connection quite quickly. (Jenny)    In our healthy eating area, we have some of the posters up and we do reflect on them during meal times. We also have incorporated a morning tea, lunch and afternoon tea poster. So the kids come with a variety of lunch boxes, but we’ve tried to focus on what foods [are] great for morning tea and why, and same with lunches, and combine that with the visuals as well to extend on that a bit further. And in our bathroom, we have our hand washing [posters], which has been great, and we used our dental posters and resources during dental week. So we actually had a display up and the children were able to do different things with that. And then we’ve read the book as well as had the book on display for the children to read themselves. And that's been reflected in the other rooms as well, especially with the hand washing and the dental side of things. I’ve definitely found it sustainable throughout each of the year level or age groups. [The posters] are definitely something that the children in our nursery room are able to [use] to make connections, just as much as our older children are. (Jenny)    I feel like those four books are like the main topics that are incorporated when it comes to hygiene and that, especially the ‘healthy teeth’ [book], because there’s so many families that say their children won’t brush their teeth and a lot of kids in this setting haven’t even been to the dentist before. That’s only a select few. So I feel like it really encouraged them with [personal hygiene]. (Davina)  You know what?… because there’s so many specific words, quotes [catchphrases], it might be useful if there was a list of them. And maybe one or two A4 pages in colour, because then we could laminate it and have it displayed in the classrooms for the teachers to remember. Do you know what I mean? So it’s not [an] out of sight, out of mind type of thing… Even if we got a list or a print‑out of that, the children can maybe decorate [their own]. We can print it out, put [it] on a big paper, and then the children can add their drawings and stuff on top of that. (Amy)    I would say ‘my healthy teeth’ ‘cause we find, in our centre, we’ve got a lot of mirrors [and] we use… costume set ups and often they like look[ing at] their own face in the mirror. So, after [reading] the book, they would look at their teeth in the mirror. So, they particularly like that one, but they enjoyed all of them as well. (Amy) |
|  | Ensuring safe and healthy learning environments | They asked, the educators, to make a story with the children [asking]. Do you know what I mean? So it was still useful in that regard, and they were still engaged. And we read all the [stories] and we wanted the children to express their feelings as well, so we can take notes and then extend on that as part of our programming in general. (Amy)    I really liked the last page where it had all the activity ideas as well. And I liked how, because a lot of the children are scared of going to the dentist and stuff like that, it showed [how to] encourage them a bit more and make it special with the special glasses and what not. The activities, the types of activities – there’s dramatic play and there’s fine motor skill – and it includes all developmental areas as well. (Amy)  Part of [it] was teaching them to recognise their own body feelings, like hunger and thirst and often, before we go to meal times, we’ll talk about how our tummy feels then, and then after eating, how our tummy feels then, and also [how] food will give you energy and what power bugs [are in their tummy’s] as well. (Amy)    [Regarding dialogic reading], even [with] the more non-verbal kids, [those] who aren’t using verbal language, it's good too for them to see a book with not lots of words. But, especially with those kids, when you can see they’re not feeling well, and things like that, you can use the books to sort of help you, with pictures, to [ask] ‘how are you feeling?’ And ‘is it something in your belly?’ (Linda)  They have been quite handy in my room, especially the ‘healthy teeth’ [book], was probably the more popular one in my room because of our dental visits. And we’ve got a couple of, with the socio-demographic of the area, we’ve got couple of [Department of Child Protection] kids that may have had teeth removed and that sort of thing, or [have] silver caps over [their teeth]. So, it sort of helps with those [children] to talk about [things]. (Shay) |
|  | Helping children to make real-life connections | We use the book as the starting point [for teaching children about a health topic]. We cut … the end of the plastic bottle. And then we gave them a pretend toothbrush [and] shaving cream or something [to symbolise toothpaste]. And [the bottle end] was like the tooth. And I'll be, like, [demonstrating] brushing teeth [with the children]. (Amy)  Having the healthy teeth book when we’ve got our dentist visits coming and looking at when the koala was [at] the dentist saying, ‘Oh, did you get this [when you saw the dentist]? Did the dentist use this [tool]? And did you lie in the chair? Did you have the glasses [on] and that sort of thing. So it was being related to what they’ve been doing [and that] was helpful. (Shay)  The tummy [book] has been quite relevant in that we have had a lot of sickness happening at kindy in the last term. That one's probably come out the most recently. And being able to have those conversations with kids… even yesterday we had a child go home with a temperature and it was a feeling in his tummy [like in the books; that helped us identify it]. (Linda)  We have actually been doing bugs and insects a lot too, last term. So there [have] been some connection[s made by children] with the outside insects and bugs [and those in] the books. (Linda)  We did a big wombat stew focus last term. We had the Australian animals and we were talking about Australian animals. So there was a connection made that, yeah, the animals in the books were Australian, but it wasn’t ‘til after we were having those conversations around wombat stew and Australian animals that… we realised that our children didn’t know a lot of animals. So ‘cause, we do have a large proportion of [children from] non-English speaking [backgrounds] as well. It’s a bit tricky too because, in a lot of cases, we’re still just learning basic words and things. So, some of those bigger concepts, which is another reason the books are good, because some of the pictures help provide the story, which gives the children, who maybe can’t understand us verbally, some context to what we’re [talking about]. (Linda)    The one thing that I found really interesting with the children, which is something I’ve never done before, is when their stomach grumbles, if they’re hungry or if they’re thirsty. It’s something I’ve never really taught them before. And then, we were reflecting on ourselves, even us [adults] sometimes we don’t know if we’re hungry or thirsty. So, it is important to teach children… We have the Munch n Move programme, and the whole point is to reduce obesity, so it was a good learning opportunity that we’ve been working [on] with the children. (Davina)    I do think of the germs going down the sink because the kids [have] really jumped on that. So now, when I’m washing my hands, I'm like, oh yeah, germs are going down. (Jenny)  The sickness one, [‘when we are sick’, it shows], everything that happens when you’re sick. It was really nice to see even the different things like the prescription because sometimes it can be a bit daunting for the kids to have their medicine and [it can] be confronting, not only going to [the doctor], but then them telling you what needs to be done, [so this] was helpful [for building their familiarity and confidence with health procedures]. (Jenny)    The turtle and the koala are big. The koala because he’s so consistently in [the books]. But I think a big topic was the wheels on the turtle. That again led us off. And that’s the best part of it. Yes, we’re talking about, I think it was in ‘when we're hungry’, but the conversation led into a whole different thing, and it was great to be able to extend on that with the older children. We got to talk about [how] sometimes people’s legs don’t work as best as they could, and sometimes they need a bit of extra help. And that was something [the turtle with wheels] could do [for us in teaching]. It made it really inclusive without a focus on there are disabled people, if that makes sense. It was just naturally part of the book. And the children picked up on it in a positive way, which is a big thing. So, definitely the turtle. (Jenny)  To be honest, they all really liked all of [the books] because they’re so familiar with Australian animals from Naidoc week, Reconciliation week and through random dramatic play, like [with] puppets [and] the children always love animals. There wasn’t a particular one that all [the children] were most drawn to [but], obviously, the koala was a main [character because it’s on the front] cover. But, no, they all really like all of [the books]. They’re all quite familiar with Australian animals already [so that helps]. (Amy)    They, the kids, really liked ‘my healthy tummy’, that was their fave. I really liked that, to be honest. I’m not sure why. I did ask them why they asked for it, 'cause they asked for this one a few times. I don’t know whether it [was] because it had picnic aspects, which we do have picnics here, and a lot of play like, you know, the visuals of the playground and different things like that. But their favourite one was the ‘my healthy tummy’. (Jenny)    We’ve done the swinging the trunks one [When we are sick] a lot. Because we’ve had a lot of sickness the last few weeks, it’s been horrific. But [when we’ve] used the swinging their trunks one, they haven't caught onto it as much. I think mainly because us, as educators, we haven't continued to model that. We have when we're reading it, but it hasn’t been something that we’ve caught onto; we’ve always said ‘cover your mouth’. So we sort of revert back to saying that faster than we remember to say ‘swing your trunk’. But we are working on it because we do really like the concept of it – the concept’s spot on. But the the main one was the hand washing – the discussions in the bathroom, making sure that they’re using soap. Because one child always turns around and says they’re using soap. And we’ve had a child cry because they thought they were washing something alive down the sink, because another child was saying, ‘Oh well, she killed the bugs off’. So we got the book out and had another read. (Jenny)  The [‘when you are sick’ book] comes into that. And because… one of the children’s just had a fall and broken their arm, I'm like, ‘she went in an ambulance’ and that sort of thing, so it’s just incorporating it [in] with what’s relevant at the moment [and explaining health procedures to children to they can better understand]. (Shay)    We [educator and children together] reflected on the books once read and they were really sharing their past stories of times when they were hungry [or], for [the book] ‘my healthy teeth’, going to the dentist. But ‘my healthy tummy’ is when they were really reflecting on times that they have been sick or their family’s been sick. It was a yellow book, the ‘when we are sick’, [and] they actually were bringing back topics like COVID and stuff. So, I guess it made us realise COVID actually really did impact the little ones. (Davina) |
|  | Enabling developmentally responsive adaptations | [The books are] really age appropriate for childcare, which is something I feel there’s not a wide variety of resources and we tend to stick to YouTube or books that are outdated. So that’s what’s great about the Little Aussie Bugs. It’s updated research, that’s age appropriate for the children. (Davina)    I hadn’t seen a programme [like the books] that really suited the earliest [years]… you see things for school and for the older kids, but around health and nutrition you don’t. I haven’t seen many [age specific resources] in my 18 years [as an educator]. The odd thing will come out here and there, but nothing specifically covering all of it together… that was really appealing to me… and that it is so open-ended, which is very much what early childhood is about. (Linda)    The PD actually engages you in that learning about dialogic reading and is so focused on the early learning side of it. (Jenny)    Especially the germ visuals, of them going down the drain, we’ve had them used in our baby’s room and… in our kindergarten room, which is 3 to 5, so far. (Jenny)  It’s been our biggest one, the healthy eating [book] as it’s [more] a visual one rather than a practise one [for our children]. But we are slowly getting there, and I know that our toddler room is looking forward to doing a bit of a healthy eating [theme] next month… It’s helpful that [the books] can go across all the levels [ensuring consistent messaging]. (Jenny)  Often we’ll use these books as a base for some of our intentional teaching practices and room setups. [For example], if we’re doing a dramatic play set up or we’ve done science experiments with… water, soap and pepper… we’ve used the books and then extended upon that with the activities. So, we’ve used the activities and things based on the book in different developmental domains, like sensory and fine motor skills. (Amy) |
|  | | |
| 3. Strengthening community action | Encouraging knowledge sharing among staff in a centre | These books and the course [were] useful for me as educational leader [both] to share my knowledge and give tips and advice [to other educators at the service] (Amy).    It’ll be nice to read it with the whole staff team and have those discussions… we’ve also had new staff, and some staff leave, so it’ll be nice to go over [the resources] with everybody. (Linda)    I don’t just hand [my colleagues] the book and say there you go… I briefed them about the purpose of this book… what we are trying to do, what are we trying to teach and how to use these books to get the most use out of them. (Amy)    It’s something that I’m really looking forward to. [Seeing] the other staff engaging with [the books and resources] as well, because it is so important for the kids. For me it was very inclusive of what I’ve [previously] learned… it was so helpful to have [the books and resources to support other educators at my centre]. (Jenny)    Once I’d completed [the PD], and I took [the books] to the staff in the rooms to let them know about [them], they knew I was doing something, but they weren’t exactly sure what.  I introduced them to the books [and] the page at the back, which they found really helpful because they were all a bit like, ‘OK, a good book to read’. (Jenny) |
|  | Supporting centre-family connections | [Regarding the] video with the hand washing, the children drew a picture of a tap and then they actually wrote [traced the] relevant words and coloured in, and we’ve laminated it for them. They’ve actually taken it home [as] a further extension of their learning. (Amy)    We went back to our policy and have sent an e-mail out to ensure that the families and children are washing their hands on pick up. We’re going to utilise the resources and add it into [the family] orientation pack. (Davina)    [We] sent an e-mail out and reflected on the Aussie bugs programme, of the importance of ensuring the families and children are washing their hands in the morning. (Davina)    I don’t know if there’s a fact sheet or something very simple [to] share with the educators or even just having a resource for the families, but something that’s not too complex… that we could hand out ‘cause I know, for the families, we always try to give them information, but sometimes, if it’s too much, they won’t read it. So [it needs to be] something that’s very simplified. (Davina)    *Interviewer: Is that beneficial for the children to share their own experiences?*  I think so and it gives us an idea of whether they are connecting when they can share something from home. It tells us that they’re comprehending, from what we're saying, and that they are connecting it to home, which is transferring knowledge... So, for us, that’s a good indicator that they’re actually taking in what you’re saying [and], you know, kids love to talk about themselves. (Linda) |
|  | Connecting centres through the online course | [The discussion boards facilitated] a little online community… already educators always talk to each other… I’m already a member of multiple Facebook groups” (Amy) - so the programme can easily become part of existing online educational conversations between colleagues.    [The discussion board within each module] allows you to expand on your thinking of what other centres are doing and what we can implement differently.” (Davina)    I definitely got things from others [in the discussion boards]. I wouldn’t say I was a person that put up something specifically meaningful, [as] I do get stuck for words sometimes. But it definitely was helpful to see other people’s responses. I took a little bit on board from that and noted it in [my reflective] journal… little things that I’ve noticed about what they had their focus on, that I hadn’t focus[ed] on. That was helpful [as it prompted self-reflection on my practice]. (Jenny) |
|  | | |
| 4. Developing personal skills | Developing the skills of educators | That's something I found quite interesting [the videos on dialogic reading] because it extended my knowledge. (Davina)  It's huge to have that knowledge [about dialogic reading], so you are incorporating that… it's not just reading a book, it's making those connections [which are meaningful and impactful for children’s learning and development]. (Jenny)  [engaging with the books] made me realise that a lot of the stuff that we read isn't exactly on par with maybe the right side of things… we've got some old books about doing this and doing that and a lot of it is quite old concepts… So that was a big thing that I noticed. There are right ways for the teaching to occur, and even if it is in a play-based environment, we have to have those supportive resources to [be] up to date.” (Jenny)  The dialogic reading… it’s huge to have that knowledge. So you are incorporating that, it's not just reading a book, it's making those connections and then being able to be like, ‘yep, we smashed out that literacy learning’ it wasn’t [just] ‘we read a book’. (Jenny) |
|  | Developing the skills of children | Especially with those [non-verbal] kids, when… you can see they're not feeling well… you can use the books to help you, with pictures, to [ask], ‘how are you feeling? Is it something in your belly? (Linda)  [talking about ‘When we are sick’] “it’s been interesting hearing the children use the terminology of ‘remember, we’ll get the bad bugs, we’ll get sick’... really interesting seeing them gain that learning from the books and transferring that [learning] to other areas. (Davina)  [the books also provide] the opportunity to the children to create their own story. I love that! That is something that you cannot do with other books. And the children were adding [to] the story as well. (Pam)  We try to allow the children to express themselves as much as they can. So, what I have been doing that I haven't done before, with the books, is that I allow the children to have a story time with their friends that they can create stories. So, they grab the same book. One day one of them can read it and then the following day it can be another [child]. And they have [each] been creating their own story… recreating some part of what I have been saying before, but they have been create[ing] their own pattern of expression [from that foundation]. (Pam)  We’re thinking of ways to do this stuff with the younger children [to] develop their skills as well. For [those] that can't walk up to the tap and do it themselves, we've got little face washes for the child [to] try to include [them]. We’ll go over it, [how] to do it properly, to wipe their own face, do their own hands as well, [and] even though it's not done perfectly, it’s still good… to at least try… laying the foundation, which is also good for their fine motor skills. (Amy)  We’re trying to teach kids that reading isn’t just reading the words – you can be a reader without having words in a book. So that’s another part of literacy we really focus on. So, having books that don’t have a story is another way to show these kids that… you don’t need words to read a book. You can pick up any book, look at the pictures and tell a story. So yeah, that has been another branch of it, the literacy side, that I really like because there aren’t [many] books that don't have… lots of words and story to them. (Linda)  When we started reading the book, the children were saying the pictures and everything, they start creating their own story. So, the first one was a little germ at the bottom of the picture… And they start saying ‘and then it goes to your body and then it goes to your tummy and then it’s when you get sick’ and then they [say], ‘oh, but we have little warriors inside of our bodies’. So they [are] creating their own story [from the pictures]. (Pam)  Those specific vocabularies [are] useful and I’ve actually gotten feedback from some of the room leaders saying that some of the children are starting to remember those wording, even just at random times, they’ll just say it. (Amy)  So I found actually sitting with the children and [saying] ‘123, healthy sticks’, because the children often repeat language as well, it’s easy for them to remember. So, the rhyming type language like that is quite useful. It’s stuck with them, even after the fact, even after eating and they’re doing something completely different there’s some child just randomly [saying it]. (Amy)  [since using the books] ... I’ve seen children automatically go and wash their hands without us having to ask them where, before… you would always have to make it make a point of doing so, *then* they'll start. But now, most of the time, they’re doing it automatically. Sometimes they’ll talk about how they do it [at] home. Or when they’ve been sick before. Or, when we’re talking about the hungry book, they’ll just talk about what their favourite food is or something like that. They talk about how it is in real life, at home, their past experiences [and feelings]. (Amy) |
|  | | |
| 5. Reorienting health services | Educators promoting preventative health practices | [Health promotion] is obviously a priority, especially post-COVID and all of that, especially health and hygiene. It’s something that’s become more important… the health and nutrition side, when we’re [dealing] with allergies and with not sharing food and all of the responsibilities we’ve got as educators [around that] as well. (Linda)    We utilised that book to educate the children on the importance of washing their hands because we found, during COVID, everyone was big on the hand sanitising, washing their hands and everything. But yeah, it’s kind of slipped a bit, so we’re just really trying, we’re having to remind the children all the time. Supervising the bathroom, washing your hands. So it’s been interesting hearing the children use the terminology of ‘remember, we’ll get the bad bugs, we’ll get sick’. Yeah, it’s just really interesting seeing them gain that learning from the books and transferring that to other areas. And it just shows that the key messages portrayed in these books, it’s age-appropriate because the children understand it. (Davina)    [During] group time, when we finish with the book, I always ask them ‘what do you think we have to do next because it’s lunchtime’. And then they say, ‘we have to go and wash our hands’ and then I ask ‘what do you need to do when you’re washing your hands’ and then they say, ‘we need to pull water, we need to put soap’. So I always try to use what they saw in the book so we can continue with the following activity. (Pam)    Get more out of what we’re doing daily. Yeah, I use [the books] quite often in our group mat sessions as reinforcement and, because we have a washing hand song before we go to mealtimes, it’s part of our transition we sing a song about… all the steps of washing your hands. So, finding ways to incorporate [the books] in there, and then we talk. So, [with] one of the books out, we’ll talk about the different coloured germs and when they wash the purple ones down the drain and that sort of thing. We use it in that sense, but not [the] activities [at the back of the book]. (Shay)    [The books are useful], when we’ve got the hospital set up… I think the tummy one’s in there at the moment. And we did have an oral health visit, a dentist exam so, you know, when that’s coming up… we’ll make sure we have [the book] around to read to the kids. (Linda) |
|  | Building confidence around health procedures | [These health topics are] very important and I really like using books and group time to introduce these ideas and talk about them and have the time with the kids to see what they know and see, as you read them or go through different things. Seeing their development of learning. So it actually was really helpful. I like having specific books, knowing what they’re for, and how to put them into my day. (Linda)    ‘Cause our kids do bring their own food and just having those conversations around healthy eating without being pushy or like saying, ‘oh, you can’t eat that’. Trying to make everybody more aware. And it’s a nice way to do it, without putting too much pressure on anybody that what’s in their lunch box isn’t good enough or they shouldn’t be eating it. With some kids [just] having food in their lunchboxes is something you gotta be [promoting as] they need to eat something. Just being able to have little conversations around [healthy eating with] some kids not even knowing what some fruits and vegetables are… it is important. (Linda)    [The course and resources] made me realise [health literacy] is essential and we’re a vital learning opportunity for them to learn about this importance because, as much as families might say ‘wash your hands’ and all the hygiene [stuff]… that’s kind of a routine thing [in the home environment]. But having an actual resource, like a book or doing certain activities is what is really going to allow them to learn the importance of it [through reinforcement outside their home environment. I now realise and can do this]. (Davina)    When they’re washing their hands and all of that, [I] role model it to them as well. ‘Cause they want [and] it’s better they see me do it as well. So, I’ve [brought] my own toothbrush from home... Not that I normally wash my teeth on my lunch break, but I [do now to help demonstrate good practice to the children]. (Amy)    In between actual structured activities… because we have to take time to set up the activities, and we’ll do a little circle time [with] a book. So, these books are easy just [to] quickly pull out and just sit with [the children] and talk about [while] someone [else] is setting up an activity. (Amy) |
